# Invasive non-typhoidal *Salmonella* infections in sub-Saharan Africa: a systematic review on antimicrobial resistance and treatment

**DOI:** 10.1186/s12916-020-01652-4

**Published:** 2020-07-17

**Authors:** Bieke Tack, Jolien Vanaenrode, Jan Y. Verbakel, Jaan Toelen, Jan Jacobs

**Affiliations:** 1 Department of Clinical Sciences, Institute of Tropical Medicine, Antwerp, Belgium; 2 Department of Microbiology, Immunology and Transplantation, KU Leuven, Leuven, Belgium; 3 Faculty of Medicine, KU Leuven, Leuven, Belgium; 4 Department of Public Health and Primary Care, KU Leuven, Leuven, Belgium; 5 Department of Development and Regeneration, KU Leuven, Leuven, Belgium; 6 Division of Woman and Child, Department of Pediatrics, University Hospitals Leuven, Leuven, Belgium

**Keywords:** Non-typhoidal *Salmonella*, Invasive infections, Sub-Saharan Africa, Antimicrobial resistance, Antimicrobial treatment

## Abstract

**Background:**

Non-typhoidal *Salmonella* (NTS) are a frequent cause of invasive infections in sub-Saharan Africa. They are frequently multidrug resistant (co-resistant to ampicillin, trimethoprim-sulfamethoxazole, and chloramphenicol), and resistance to third-generation cephalosporin and fluoroquinolone non-susceptibility have been reported. Third-generation cephalosporins and fluoroquinolones are often used to treat invasive NTS infections, but azithromycin might be an alternative. However, data on antibiotic treatment efficacy in invasive NTS infections are lacking. In this study, we aimed to assess the spatiotemporal distribution of antimicrobial resistance in invasive NTS infections in sub-Saharan Africa and to describe the available evidence and recommendations on antimicrobial treatment.

**Methods:**

We conducted a systematic review of all available literature on antimicrobial resistance and treatment in invasive NTS infections. We performed a random effects meta-analysis to assess the temporal distribution of multidrug resistance, third-generation cephalosporin resistance, and fluoroquinolone non-susceptibility. We mapped these data to assess the spatial distribution. We provided a narrative synthesis of the available evidence and recommendations on antimicrobial treatment.

**Results:**

Since 2001, multidrug resistance was observed in 75% of NTS isolates from all sub-Saharan African regions (95% confidence interval, 70–80% and 65–84%). Third-generation cephalosporin resistance emerged in all sub-Saharan African regions and was present in 5% (95% confidence interval, 1–10%) after 2010. Fluoroquinolone non-susceptibility emerged in all sub-Saharan African regions but did not increase over time. Azithromycin resistance was reported in DR Congo. There were no reports on carbapenem resistance. We did not find high-quality evidence on the efficacy of antimicrobial treatment. There were no supranational guidelines. The “Access group” antibiotics ampicillin, trimethoprim-sulfamethoxazole, and chloramphenicol and “Watch group” antibiotics ceftriaxone, cefotaxime, and ciprofloxacin were recommended as the first-choice antibiotics in national guidelines or reviews. These also recommended (a switch to) oral fluoroquinolones or azithromycin.

**Conclusions:**

In addition to the widespread multidrug resistance in invasive NTS infections in sub-Saharan Africa, resistance to third-generation cephalosporins and fluoroquinolone non-susceptibility was present in all regions. There was a lack of data on the efficacy of antimicrobial treatment in these infections, and supranational evidence-based guidelines were absent.

## Background

Globally, non-typhoidal *Salmonella* (NTS) are an important cause of foodborne and self-limiting enteritis [1]. In sub-Saharan Africa, however, NTS frequently invade normally sterile sites and cause invasive infections [2]. Most of these invasive NTS infections are bloodstream infections, but also meningitis and other focal infections occur [3]. In bloodstream infections, NTS is one of the three most isolated pathogens in sub-Saharan Africa [4]. In addition, in 2017, it was estimated that 79% of the 535,000 global cases of invasive NTS infection occurred in this region. Moreover, 85% of the estimated 77,500 deaths worldwide occurred in sub-Saharan Africa, corresponding to a high case fatality ratio of 15.8% [1]. Most invasive NTS infections occur in susceptible hosts, i.e., HIV-infected individuals or young children with comorbidities like *Plasmodium falciparum* malaria infection, anemia, or malnutrition [5]. Most infections are caused by *Salmonella enterica* subspecies *enterica* (hereafter *Salmonella*) serovars Typhimurium and Enteritidis [4]. For both serovars, the strains circulating in sub-Saharan Africa have different genotypes than the ones circulating in high-income countries. The sub-Saharan African strains have a more human-adapted and more virulent genotype and phenotype. In addition, they frequently carry antimicrobial resistance (AMR) genes [3, 6].

Multidrug resistance (MDR), i.e., co-existing resistance to ampicillin, trimethoprim-sulfamethoxazole, and chloramphenicol, is currently widespread among NTS in sub-Saharan Africa [3]. All three antibiotics are categorized by the World Health Organization (WHO) model list of essential medicines as “Access” antibiotics and thus recommended as an empiric first-choice treatment for many infectious syndromes, including invasive infections [7, 8]. Alarmingly, third-generation cephalosporin (C3G) resistance and fluoroquinolone non-susceptibility (FQNS) have now been reported in various countries in sub-Saharan Africa [3]. Both antibiotic classes are listed as “Watch” antibiotics, because they are considered as critically important antibiotics for human medicine [9] and they are at high risk of selecting bacterial resistance. As such, they are prioritized as key targets of stewardship programs and monitoring [7, 8].

The WHO Global Action Plan against Antimicrobial Resistance highlights both the importance of strengthening the current knowledge through surveillance and optimizing the use of antibiotics [10]. Firstly, although many studies report surveillance data on the presence of MDR, C3G resistance, and FQNS, these data have never been clustered to assess their temporal evolution and spatial distribution in sub-Saharan Africa. Secondly, the optimal use of antibiotics to treat invasive NTS infections is unknown. To the best of our knowledge, no supranational guidelines on the antimicrobial treatment of invasive NTS infections exist, and no data on the efficacy of antimicrobial therapy in invasive NTS infections are available from interventional or high-quality observational studies. As a consequence, clinicians currently use antimicrobial regimens that are mimicked from antimicrobial regimens for typhoid fever [5].

In this systematic review, we aim to assess the temporal and spatial distribution of AMR in invasive NTS infections in sub-Saharan Africa. We focus on MDR, C3G resistance, and FQNS, as these cover the main antibiotics used to treat invasive NTS infections. Secondly, we collect and describe all available evidence on antimicrobial treatment efficacy and safety in invasive NTS infections. Finally, we collect and summarize the recommendations regarding the antimicrobial treatment of invasive NTS infections published in research or review articles and supranational or national guidelines.

## Methods

### Search strategy and study selection

We conducted a systematic review of the literature according to the PRISMA guidelines (Additional File 1: Table S1). The study protocol has been publicly registered at study initiation (PROSPERO, CRD42019137673). We searched for all studies published from inception until 29 January 2020 on four databases: MEDLINE, Ovid Embase, African Journals Online, and African Index Medicus. The search strategy was developed together with a biomedical librarian (MJ).

For the first research question on the temporal and spatial distribution of AMR in invasive NTS infections in sub-Saharan Africa, we used the following search concepts: “antimicrobial resistance” and “non-typhoidal *Salmonella*” and “invasive infection” and “sub-Saharan Africa”. For the second research question on the available recommendations and therapeutic efficacy data, the search concepts were as follows: “antimicrobial treatment” and “non-typhoidal *Salmonella*” and “invasive infection”. The full search strategies for both review questions in MEDLINE and Ovid Embase are provided in Additional File 1: List S2. We used adapted versions of these strategies in the two African databases. We completed our search by scanning the reference lists of review articles encountered during the search.

Only full-text articles or guidelines were considered as eligible, i.e., non-full text conference proceedings or book chapters were excluded. Only studies published in English, French, Spanish, Portuguese, and Dutch were eligible for inclusion. Two independent reviewers (BT, JVA) screened the articles for eligibility according to the in- and exclusion criteria mentioned in Additional File 1: Table S3. The screening was performed in Qatar Computing Research Institute (QCRI, Doha, Qatar). Any conflict was resolved by a discussion.

### Data extraction

For review question 1, we extracted the study metadata, methods, and results from antibiotic susceptibility testing (number of tested NTS and number of resistant NTS or proportion of resistant NTS). For review question 2, we extracted clinical outcomes and adverse event data from interventional and observational studies and documented the antimicrobial treatment recommendations from guidelines and reviews. The data were extracted by one reviewer (JVA) and checked by a second reviewer (BT). All extracted data were compiled in an Excel database (Microsoft, Redmond, WA, USA) (Additional File 2).

### Risk of bias assessment

The risk of bias was assessed by one reviewer (JVA) and checked by a second reviewer (BT) (Additional File 3). For research question 1, the MICRO checklist [11] and the Joanna Briggs Institute Critical Appraisal Checklist for Studies Reporting Prevalence Data [12] were used. Based on the grading system described in the MICRO checklist, the quality of each study was graded with “A” being the highest quality and “E” being the lowest quality grade.

For research question 2, we used the Joanna Briggs Institute Critical Appraisal Checklist for Quasi-Experimental Studies (non-randomized experimental studies) [13] for studies reporting therapeutic efficacy. For guidelines, we used the AGREE II checklist [14], and for recommendations in expert opinion papers or reviews, the Joanna Briggs Institute Critical Appraisal Checklist for Text and Opinion Papers [15].

### Definitions

**Fig. 1:**
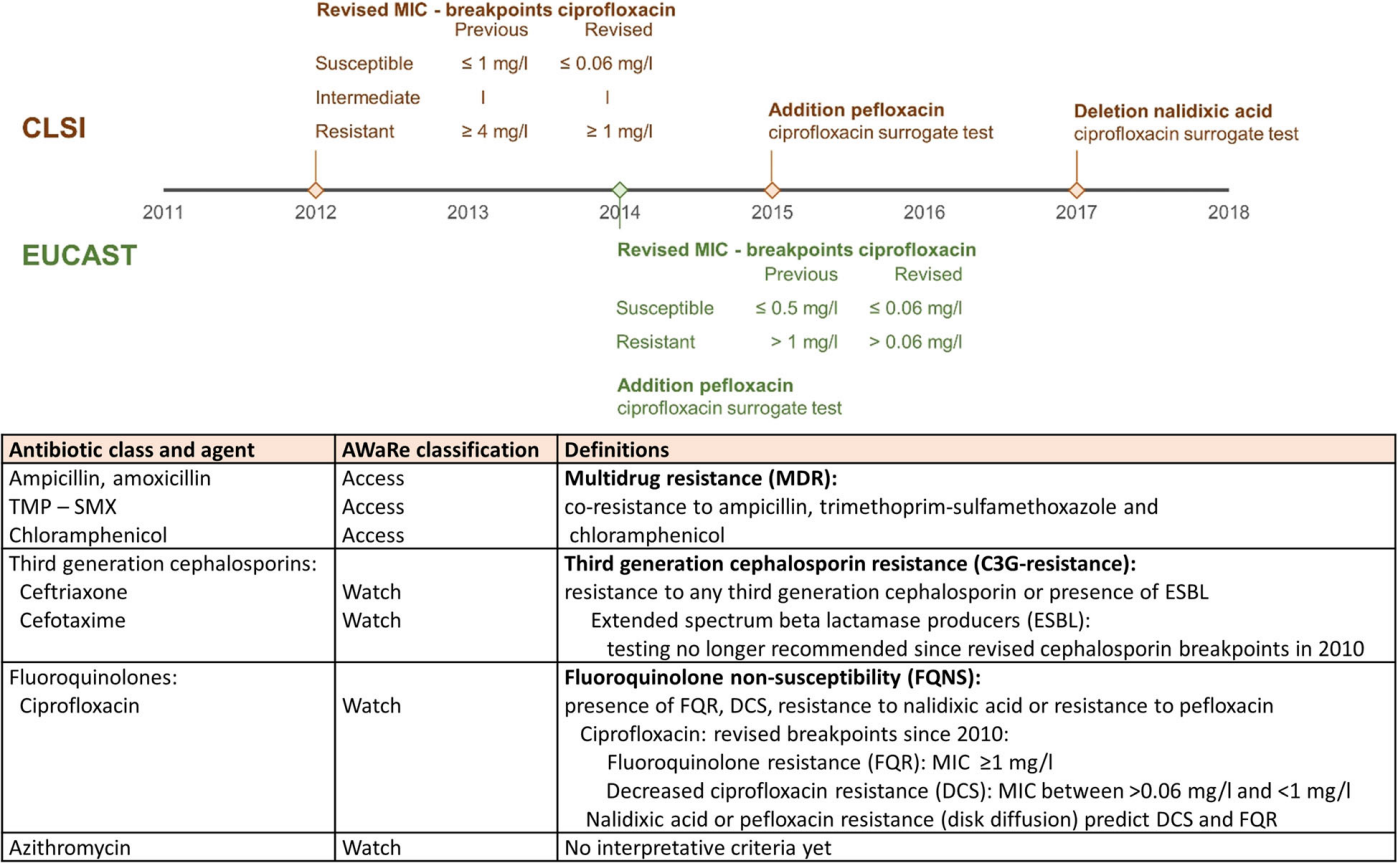
Overview of the definitions used in this review and of the changes in recommendations for fluoroquinolone susceptibility testing over time. A thin line represents the MIC—range of the intermediate susceptibility category according to CLSI (brown) [18, 19]. There is no intermediate ciprofloxacin susceptibility category defined by EUCAST (green) [22]. The antibiotic agents are classified according to antibiotic class and the AWaRe classification. The latter is defined in the WHO Essential Medicines List and categorizes antibiotics into Access, Watch, or Reserve group antibiotics [7, 8]. TMP–SMX, trimethoprim–sulfamethoxazole; MIC, minimum inhibitory concentration; CLSI, Clinical and Laboratory Standards Institute; EUCAST, European Committee on Antimicrobial Susceptibility Testing

Countries were assigned to the geographic regions identified by the United Nations [16]. We considered isolates as resistant when they were reported as resistant or “intermediately resistant” or “intermediately susceptible” or “non-susceptible” [17]. Results for ampicillin and amoxicillin were aggregated. Figure 1 gives an overview of the definitions of MDR [5, 23, 24], C3G resistance, and FQNS [18, 22, 24, 25] used in this review.

For C3G resistance and FQNS, we pooled the data as such that the maximal number of resistant NTS was obtained. Over time, the guidelines for fluoroquinolone susceptibility testing have changed (Fig. 1). These revisions were driven by reports about treatment failure in patients with invasive *Salmonella* infections. *Salmonella*, mainly Typhi, isolated from these infections with clinical failure of ciprofloxacin were reported as ciprofloxacin susceptible but demonstrated resistance to nalidixic acid or a moderately increased ciprofloxacin minimum inhibitory concentration (MIC) value [26, 27]. Ever since, it is discouraged to use fluoroquinolones to treat *Salmonella* with decreased ciprofloxacin susceptibility (DCS; Fig. 1) [18, 19]. In addition, the use of nalidixic acid as surrogate disk testing is no longer recommended since 2017 due to failure of detection of *gyrB* and plasmid-mediated fluoroquinolone non-susceptibility [18, 20, 21, 25].

### Data analysis

For research question 1, we performed a meta-analysis to assess the temporal evolution of MDR, C3G resistance, or FQNS and created a simplified map to assess their spatial distribution in sub-Saharan Africa.

For the meta-analysis about AMR, studies reporting antibiotic susceptibility testing from < 50 NTS isolates and studies that reported only on resistant isolates were excluded. In addition, we excluded studies with grade E quality according to the MICRO checklist [11], due to uncertainty or inconsistency in their reported data. To calculate the pooled proportions of MDR, C3G resistance, and FQNS in invasive NTS isolates, we used a random effects restricted likelihood model after double arcsine transformation. Heterogeneity was assessed based on the parameters *I*^2^, *τ*^2^, and *Q*. We conducted a sensitivity analysis to assess the effect of the exclusion of studies reporting antibiotic susceptibilities of < 50 NTS isolates and the inclusion of studies with grade D quality.

To assess the temporal evolution, the meta-analysis was repeated per time period. Based on their midyear, which was rounded to the most recent year, all studies were classified into four time periods: until 1990, 1990–2000, 2001–2010, and after 2010. Data were disaggregated per period wherever possible. In addition, we performed a subgroup analysis for *Salmonella* Typhimurium and *Salmonella* Enteritidis with data from studies that reported ≥ 10 isolates per serotype. Finally, we performed a meta-regression analysis using a mixed-effect model to assess if part of the residual heterogeneity could be explained by differences in study characteristics. The meta-analysis was done in R version 3.6 in the “metafor” package (functions escalc(), rma(), predict(), print(), forest()).

Secondly, to assess the spatial distribution of MDR, C3G resistance, and FQNS, we generated a map in PowerPoint Office 16 (Microsoft). For countries without exact data on MDR, we plotted the highest possible proportion of MDR, which was based on the antibiotic (ampicillin, trimethoprim-sulfamethoxazole, or chloramphenicol) with the lowest proportion of resistance.

## Results

### Antimicrobial resistance in invasive NTS infections in sub-Saharan Africa

#### Study inclusion and characteristics

**Fig. 2:**
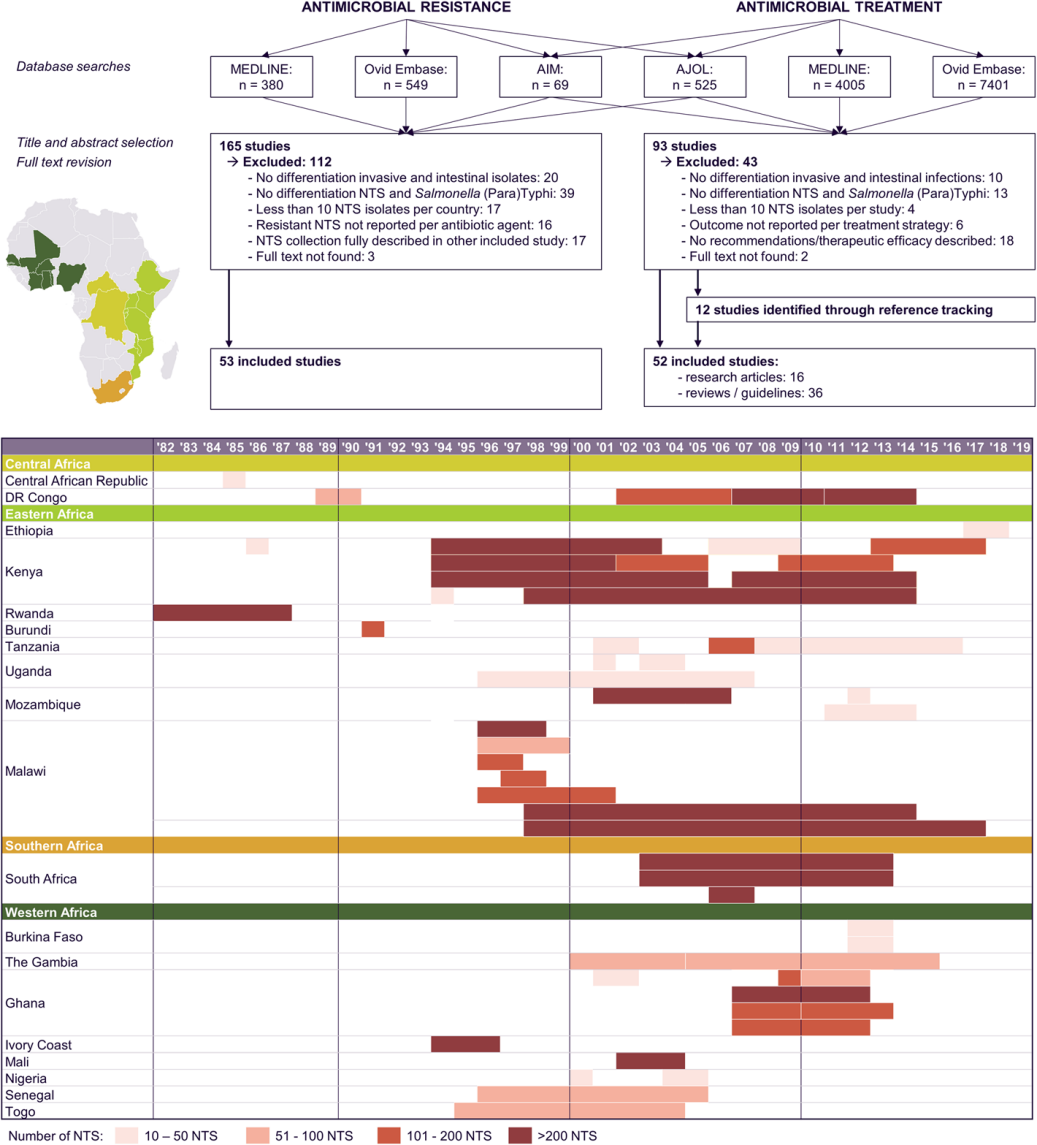
PRISMA flowchart and overview of the study selection according to the sub-Saharan African region according to the United Nations, country, and study period. All countries from which studies with data on antimicrobial resistance were included and are represented on the map. Their color corresponds to the sub-Saharan African region to which they belong according to the United Nations. No studies were included from countries presented in gray. NTS, non-typhoidal *Salmonella*; years ‘90–‘19, years 1990–2019

We included 53 studies that reported AMR in invasive NTS infections in sub-Saharan Africa (Fig. 2). The studies for which no full text could be retrieved are listed in Additional File 1: List S4. Most studies reported NTS obtained from 2000 onwards, and all four regions of sub-Saharan Africa were represented (Fig. 2). Most studies were hospital-based (40/48) and reported data from blood culture isolates only (43/53; Table 1). The interpretative criteria for the antibiotic susceptibility tests were reported in 74% (39/53), and the version of these criteria was specified in 36 studies. From the 38 studies that reported data on FQNS, only 21% described DCS and FQR separately. More than half of the studies were considered as grade D quality (31/53). We considered four studies as grade E quality due to uncertainty or inconsistencies in the data reported [28–31].

**Table 1:** Study characteristics of the included studies reporting antimicrobial resistance in invasive NTS infections in sub-Saharan Africa

Study characteristics - population	n (%)	Study characteristics - microbiology	n (%)
**Region sub-Saharan Africa**	***n*= 53**	**Number of NTS isolates**	**53**
Central Africa	5 (9)	10 - 49	15 (28)
Eastern Africa	31 (58)	50 - 99	8 (15)
Southern Africa	3 (6)	100 - 199	12 (23)
Western Africa	16 (30)	200 - 499	12 (23)
		> 500	6 (11)
**Study design**	***n*= 53**		
Retrospective	21 (40)	**Clinical specimen**	**53**
Prospective	32 (60)	Blood	43 (81)
		CSF	2 (4)
**Duration study period**	***n*= 53**	Blood + CSF	4 (8)
<1 year	8 (15)	Blood + CSF + other normally sterile body sites	4 (8)
1 - 2 years	11 (21)		
2 - 5 years	14 (26)	**Method antibiotic susceptibility testing**	**52**
5 - 10 years	8 (15)	Disk diffusion	28 (54)
> 10 years	12 (23)	E-test	1 (2)
		Automated methods	2 (4)
**Study setting**	***n*= 48**	Disk diffusion + E-test	17 (33)
Population based	3 (6)	Automated methods + disk diffusion / E-test	4 (8)
District hospital	19 (40)		
University / tertiary care hospital	12 (25)	**International guidelines for interpretation AST**	**39**
Multicenter	14 (29)	CLSI / NCCLS	32 (82)
		EUCAST	2 (5)
**Study population**	***n*= 50**	National guidelines (French microbiological society or BSAC)	5 (13)
Children	28 (56)		
Adults	8 (16)	Version of guidelines specified	36 (92)
Children and adults	14 (28)		
		**Definitions of fluoroquinolone non-susceptibility**	**38**
**HIV prevalence**	***n*= 26**	Mixed reporting of resistance & decreased susceptibility	10 (26)
≤20% in enrolled patients / patients with invasive NTS infection	9 (35)	Resistance & decreased susceptibility separately reported	8 (21)
>20% in enrolled patients / patients with invasive NTS infection	17 (65)	Assessed before introduction of revised breakpoints	20 (53)
**Number of screened cultures**	***n*= 53**	**Assessment of fluoroquinolone non-susceptibility**	**38**
0 - 99	5 (9)	Only nalidixic acid resistance reported	1 (3)
100 - 499	10 (19)	Only ciprofloxacin resistance reported	25 (66)
500 - 999	9 (17)	Nalidixic acid and ciprofloxacin resistance reported	11 (29)
1000 - 5000	12 (23)	Pefloxacin resistance reported	1 (3)
> 5000	17 (32)		

*NTS* non-typhoidal *Salmonella*, *CSF* cerebrospinal fluid, *AST* antibiotic susceptibility testing, *CLSI* Clinical and Laboratory Standards Institute, *EUCAST* European Committee on Antimicrobial Susceptibility Testing, *BSAC* British Society of Antimicrobial Chemotherapy

#### Multidrug resistance

**Fig. 3:**
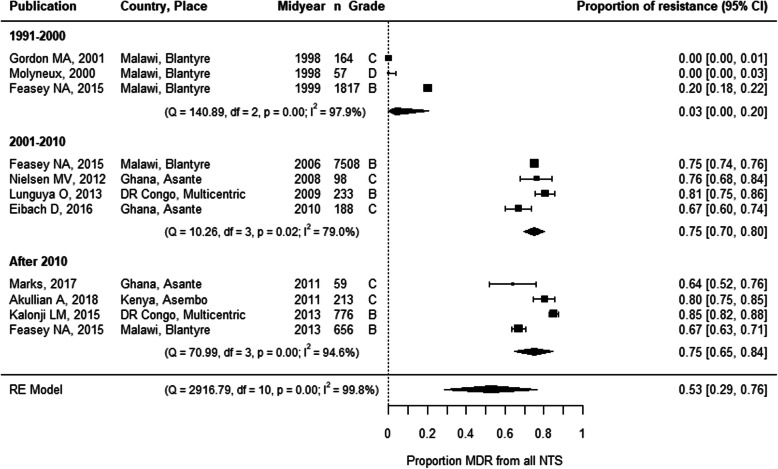
Forest plot of multidrug resistance in invasive NTS infections in sub-Saharan Africa. Each publication is identified by its first author and year of publication. Studies are ranked by the midyear of the study period during which the NTS were isolated. The grade represents the study quality and was assessed based on the MICRO checklist [11]. MDR, multidrug resistance; NTS, non-typhoidal *Salmonella*; RE model, random effects model; df, degrees of freedom; 95% CI, 95% confidence interval

**Fig. 4:**
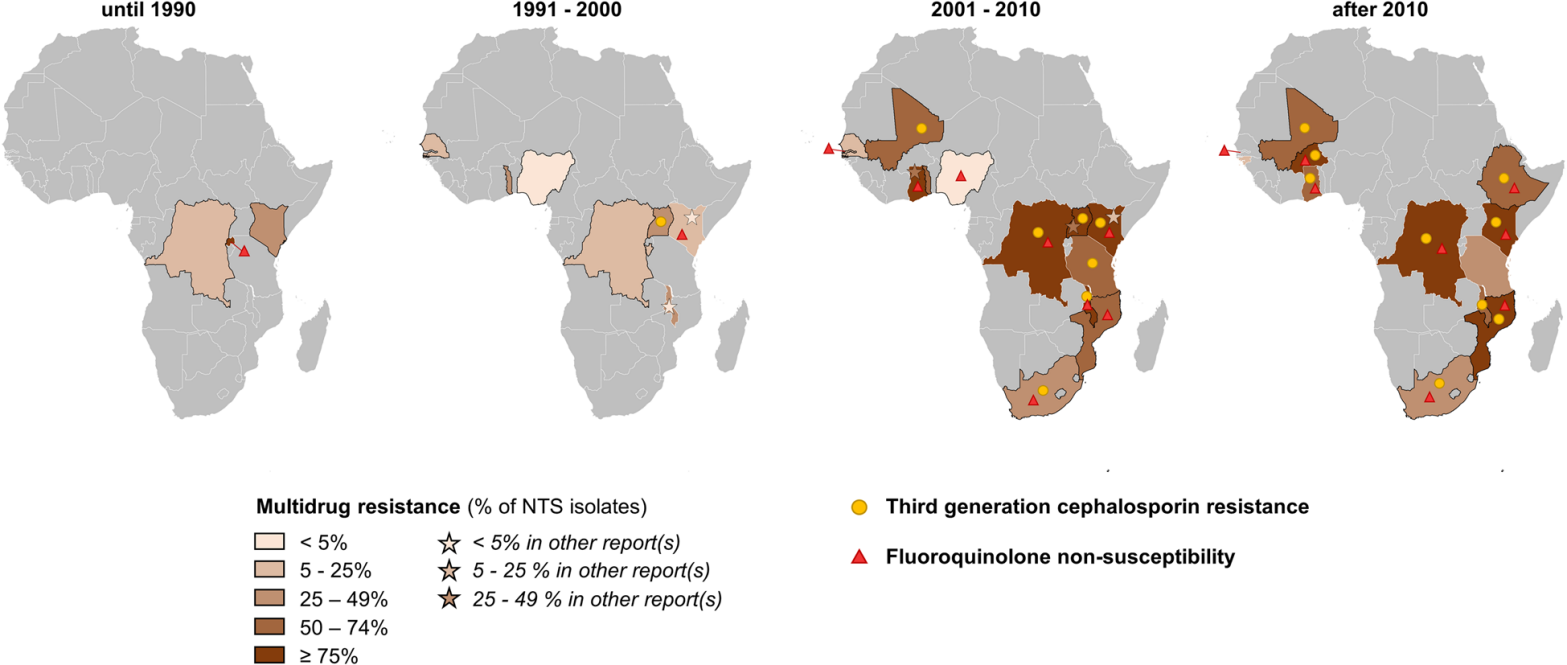
Spatiotemporal overview of the emergence of multidrug resistance, third-generation cephalosporin resistance, and fluoroquinolone non-susceptibility in invasive NTS infections in sub-Saharan Africa. The proportions of multidrug-resistant NTS per time period per country were represented by the choropleth. The presence of third-generation cephalosporin resistance or fluoroquinolone non-susceptibility was indicated with a circle or a star, respectively. For countries represented with black boundaries, the maximum proportion of multidrug-resistant NTS was plotted as no data on multidrug resistance were reported. The maximum proportion of multidrug-resistant NTS was determined as the lowest proportion among ampicillin, trimethoprim-sulfamethoxazole, and chloramphenicol resistance. For the sub-Saharan African countries in gray, no data were available. From 2001 to 2010, the maximum proportion of multidrug resistance in The Gambia was 19.4%, which is not clearly visible due to the small size of the country. For Rwanda in the period until 1990 and for The Gambia in the periods 2001–2010 and after 2010, the triangle indicating the presence of fluoroquinolone non-susceptibility is displayed right next to the respective country and connected to it with a thin line due to the small country sizes. NTS, non-typhoidal *Salmonella*

The first report of MDR NTS, published in 1990, described 246 MDR NTS obtained from hospital admitted children in Rwanda between 1982 and 1987. These 246 MDR NTS were also resistant to nalidixic acid, but still susceptible to C3G [32]. The first study that reported the proportion of MDR according to the current definition of MDR for NTS [5, 23] was published in 2012 [33]. Ever since, ten other studies reported the proportion of MDR NTS isolates (Figs. 3 and 4) [34–42]. In addition, the absence of chloramphenicol resistance allowed to deduct the absence of MDR in two studies published in 2000–2001 [43, 44].

A meta-analysis revealed an increase in MDR after 2000, with a pooled MDR proportion of 3% in the 1990s versus 75% afterwards (Fig. 3). These estimates were not impacted by the inclusion of grade D quality studies (Additional File 1: Figure S5). The emergence of MDR after 2000 was observed both in *Salmonella* Typhimurium and in *Salmonella* Enteritidis. Although data were scarce, MDR proportions in *Salmonella* Typhimurium were higher than in *Salmonella* Enteritidis after 2000. (Additional File 1: Figure S6A). Meta-regression only identified the study period as a significant moderator (Additional File 1: Table S7). Although no data from Southern Africa were included in the meta-analysis, the maximum MDR proportions, plotted in Fig. 4, suggested the emergence of MDR in all four regions of sub-Saharan Africa.

#### Third-generation cephalosporin resistance

The first three NTS strains with C3G resistance were isolated in 1994 in Kenya and were resistant to ceftazidime [45]. Until 2000, C3G resistance was absent in all nine other studies that reported results from C3G testing [30, 46–54]. Since 2001, C3G resistance was reported in 17 [28, 29, 34, 35, 40, 42, 49, 50, 54–62] of the 33 studies that reported results from C3G testing [28, 29, 33–35, 37–40, 42, 48–69]. Most (*n* = 24) studies reported data on ceftriaxone susceptibility, whereas cefotaxime susceptibility was reported in ten studies, ceftazidime susceptibility in six studies, and ESBL NTS in 10 studies (Dataset S3).

**Fig. 5:**
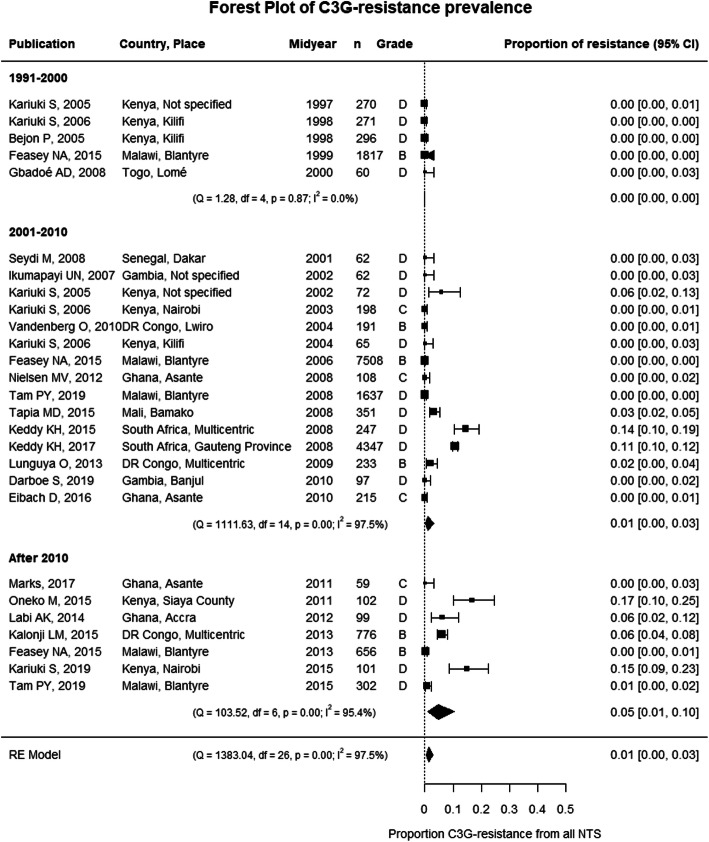
Forest plot of third-generation cephalosporin resistance in invasive NTS infections in sub-Saharan Africa. Each publication is identified by its first author and year of publication. Studies are ranked by the midyear of the study period during which the NTS were isolated. The grade represents the study quality and was assessed based on the MICRO checklist [11]. C3G resistance, third-generation cephalosporin resistance; NTS, non-typhoidal *Salmonella*; RE model, random effects model; df, degrees of freedom; 95% CI, 95% confidence interval

A meta-analysis revealed a modest increase in the pooled proportion of C3G-resistant NTS after 2010 up to 5% (Fig. 5). After exclusion of grade D studies in the sensitivity analysis, the pooled proportion after 2010 was 1% (95% confidence interval (CI), 0–4%) and did not indicate an increase (Additional File 1: Figure S5). Overall the pooled proportions of C3G resistance were similar in *Salmonella* Typhimurium and *Salmonella* Enteritidis (Additional File 1: Figure S6B), although local differences occurred [34, 56, 58, 70]. Meta-regression only identified the study period as a significant moderator (Additional File 1: Table S7). The emergence of C3G resistance was reported in all four sub-Saharan African regions since 2001 (Fig. 4).

#### Fluoroquinolone non-susceptibility

Before 2001, FQNS was first reported in NTS obtained from Rwanda between 1982 and 1987. These NTS were nalidixic acid resistant, but ofloxacin and norfloxacin susceptible; ciprofloxacin was not tested [32]. FQNS in NTS was found in one other study (Kenya, isolates obtained between 1997 and 2000). These NTS (*n* = 14) were probably DCS, as they were nalidixic acid resistant but ciprofloxacin susceptible according to the old breakpoints [48]. All other studies reported 100% fluoroquinolone susceptibility: one study tested nalidixic acid [51], eight tested ciprofloxacin [36, 46, 47, 50, 52–54, 59], one pefloxacin [31], and two ofloxacin [30, 45].

After 2000, 36 studies reported ciprofloxacin susceptibility data [29, 33–42, 46–48, 50–54, 56–69, 71–73], and 11 of them also reported on nalidixic acid susceptibility [35, 38, 42, 48, 51, 61, 65–67, 72]. Ten studies reported FQNS isolates without differentiation between DCS and FQR, although they were published after the revision of the ciprofloxacin breakpoints in 2012 [33, 37, 40, 56, 57, 59, 60, 62, 68, 69]. From the eight studies that defined DCS [34–36, 39, 41, 42, 58, 61], DCS NTS were observed in six studies (DR Congo, Burkina Faso, Ghana, South Africa, and Ethiopia) [34, 35, 41, 42, 58, 61]. FQR was sporadically observed in Nigeria, DR Congo, Kenya, Ethiopia, Malawi, and South Africa [36, 38, 58, 61, 66, 71, 72]. Both DCS and FQR were observed in South Africa and Ethiopia. In South Africa, there was more DCS (26%, *n* = 1137) than FQR (2%, *n* = 86) [58]. In Ethiopia, FQR (4/17 NTS) was more prevalent than DCS (1/17), although the reported 10/17 nalidixic acid-resistant isolates suggested more FQNS NTS [61]. Nalidixic acid resistance also suggested DCS NTS in Kenya, DR Congo, and Mozambique [38, 48, 65, 72]. However, in another study in Mozambique, nalidixic acid only detected 1/4 FQNS NTS [67].

**Fig. 6:**
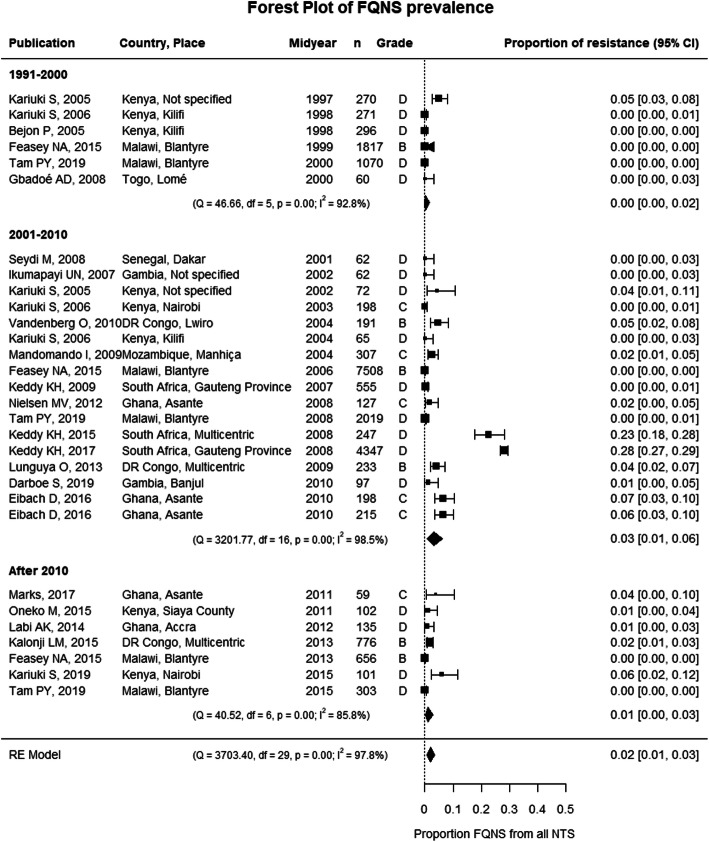
Forest plot of fluoroquinolone non-susceptibility in invasive NTS infections in sub-Saharan Africa. Each publication is identified by its first author and year of publication. Studies are ranked by the midyear of the study period during which the NTS were isolated. The grade represents the study quality and was assessed based on the MICRO checklist [11]. FQNS, fluoroquinolone non-susceptibility; NTS, non-typhoidal *Salmonella*; RE model, random effects model; df, degrees of freedom; 95% CI, 95% confidence interval

Meta-analysis showed the emergence of FQNS, but there was no clear increase in pooled proportions of FQNS over time (Fig. 6). Similar results were obtained if grade D studies were excluded. However, when studies with < 50 NTS were not excluded in the sensitivity analysis, the pooled proportions increased, albeit non-significantly, from 0.1% (95% CI, 0–1.3%) in the period 1991–2000 to 2.8% (95% CI, 0.9–5.6%) in 2001–2010 and 3.6% (95% CI, 0.2–9.6%) after 2010 (Additional File 1: Figure S5). The emergence of FQNS was similar in *Salmonella* Typhimurium and *Salmonella* Enteritidis (Additional File 1: Figure S6C). No significant moderators were identified by meta-regression (Additional File 1: Table S7). FQNS NTS were reported from all four sub-Saharan African regions in the last two decades (Fig. 4).

#### Susceptibility to alternative antibiotics

Three studies reported azithromycin susceptibility, which was interpreted according to the epidemiological cutoff for *Salmonella* Typhi (MIC > 16 mg/l). Two of them reported on DR Congo were azithromycin resistance that was found in 3.3% (*n* = 6) of *Salmonella* Typhimurium between 2007 and 2010 and in 12.7% (*n* = 49) of *Salmonella* Typhimurium between 2011 and 2015. There was no azithromycin resistance in *Salmonella* Enteritidis [34, 35]. In Burkina Faso, no azithromycin resistance was observed [42]. None of the four studies that reported carbapenem susceptibility detected carbapenem-resistant isolates [34, 40, 49, 57]. Two studies reported 100% aztreonam susceptibility [32, 53], and one study, 100% colistin susceptibility [66]. Multiple studies reported susceptibility data for antibiotics which are ineffective in vivo, i.e., first- or second-generation cephalosporins (*n* = 12), aminoglycosides (*n* = 28), or tetracyclines (*n* = 24) (Additional File 2).

#### Combined resistance patterns

Two studies from DR Congo reported NTS that were MDR and DCS or MDR and C3G-resistant (ESBL). MDR in combination with DCS was found in eight *Salmonella* Typhimurium from 2007 to 2010 [35] and in seven *Salmonella* Typhimurium and two *Salmonella* Enteritidis from 2011 to 2014 [34]. MDR and ESBL combined were found in 45 *Salmonella* Typhimurium, from which 44 were also azithromycin resistant [34]. One *Salmonella* Typhimurium was MDR, DCS, ESBL, and azithromycin resistant [35].

### Evidence and recommendations on antimicrobial therapy in invasive NTS infections

#### Study characteristics

We identified 16 primary research studies with data on antimicrobial treatment efficacy, all were non-interventional and appraised as low quality (Fig. 2). Nine of them were conducted in sub-Saharan Africa [32, 43, 74–80]. Five studies had a comparative design, comparing two different treatment regimens (*n* = 1), different durations of antimicrobial treatment (*n* = 2), and cefotaxime versus no cefotaxime (*n* = 2) (Table 2) [32, 77, 80, 87, 88]. None of the studies assessed the duration of fecal carriage.

**Table 2:** Table summarizing the evidence on the efficacy of antimicrobial treatment in invasive non-typhoidal *Salmonella* infections

Study (first author, year of publication, study site, study period, study design)	Population (age category, sample size, infection site, comorbidities)	Therapy (antibiotic agent, dose, administration route, duration, control/comparison group)	Outcome (in-hospital and post-discharge case fatality, fever clearance, microbiological clearance, recurrence, sequelae)
Chloramphenicol			
Aubry, 1992 [74], 1991, Burundi, retrospective cohort study	Adults, *n* = 69, all BSI, HIV positivity in 86/103 (83%)	Chloramphenicol	Fever clearance in 72 h: 59/69 (85%)
Molyneux, 2000 [43], Malawi, 1996–1999, prospective cohort study	Children, *n* = 57, all meningitis, clinical suspicion of AIDS in 16%	Chloramphenicol IV until defervescence and able to swallow, then PO and stop after 2–3 weeks oral treatment	In-hospital case fatality: 28/57 (49%) Post-discharge case fatality: 5/29 (17%) Recurrences: 2/29 (7%)
Graham, 2000 [81], Malawi, 1996–1998, retrospective cohort study	Children, *n* = 248, all BSI, clinical suspicion of AIDS in 16%	Chloramphenicol IV at least 5 days	In-hospital case fatality: 59/248 (23.8%)
Gordon, 2003 [76], Malawi, period not specified, prospective cohort study	Adults, *n* = 100, all BSI, all HIV patients	Chloramphenicol 2 g/day in 4 doses/day for 14 days	In-hospital case fatality: 47/100 (47%) Post-discharge case fatality: 5/19 (26%) Recurrence: 19/44 (43%)
Cephalosporins			
De Carvalho, 1982 [82], setting and period not specified, prospective cohort study	Children and adults, *n* = 11, all BSI	Cefamandole IV/IM 60–240 mg/kg/day for 12 days (longer if persistent bacteremia)	Persistent bacteremia after 12 days: 4/11 (36%)
Soe, 1987 [83], the USA, period not specified, retrospective cohort study	Children and adults, *n* = 12, 9 BSI, 2 meningitis, 1 focal infection, sickle cell disease in 1/12, AIDS in 1/12, leukemia in 1/12	Cefotaxime IV in 9/12 for 5–28 days Adults: 2–3 g/day in 3–6 doses/day Children: 100–200 mg/kg/day in 4 doses/day Ceftizoxime IV in 1/12 for 19 days Cefotaxime IV 2 days + change to ceftazidime IV 14 days in 1/12 Cefotaxime IV 2 days + change to cotrimoxazole PO 3 days in 1/12	In-hospital case fatality: 0/12 (0%) Fever clearance: median 3 days (range 1–17 days) Recurrences: 1/12 (8%)
Lepage, 1990 [32], Rwanda, 1982–1987, retrospective cohort study	Children, *n* = 246, 220 BSI, 12 meningitis, 13 focal infections, severe acute malnutrition in 23%, malaria in 11%, severe anemia in 10%	Cefotaxime IV 100 mg/kg/day (200 mg/kg/day if meningitis) for 8 days to 6 weeks (depending on the infection site and severity) Control group (*n* = 87/246): no cefotaxime because no cefotaxime available/death before blood culture results	In-hospital case fatality: 16/152 (11%) Recurrence: 4% of NTS BSI Fever clearance: mean 2.3 days (range 0.5–7.5 days)* Control group: in-hospital case fatality 64/87 (74%)
Wang, 1996 [84], Taiwan, 1990–1994, case series	Adults (> 65 years), *n* = 12, all mycotic aneurysms	Ceftriaxone (+ surgical intervention in 11/12 patients) for 23–40 days in survivors	In-hospital case fatality: 6/12 (50%) Recurrence: 0/6 (0%)
Chiu, 2006 [85], Taiwan, 1999–2003, retrospective cohort study	Children, *n* = 27, all BSI	Ceftriaxone IV in 25/27; cefixime PO in 2/27	In-hospital case fatality: 1/27 (4%): a leukemic patient with spondylitis and splenic abscess treated with ceftriaxone
Fluoroquinolones			
Cheesbrough, 1991 [78], DR Congo, period not specified, prospective cohort study	Children, *n* = 31, 29 BSI, 4 arthritis (including 2 with BSI)	Ciprofloxacin PO 20 mg/kg/day in 2 doses/day	In-hospital case fatality: 1/31 (3%) Post-discharge case fatality: 0/30 (0%) Microbiological clearance after 48–72 h: 0/30 (0%) Recurrence: 0/30 (0%)
Forrest, 2009 [86], USA, 2002–2006, retrospective cohort study	Adults, *n* = 16, all BSI, all HIV patients	Quinolones in 15/16 patients for 10–300 days (median 28 days)	In-hospital case fatality: 1/15 (7%) Recurrences: 4/15 (27%)
Gordon, 2010 [79], Malawi, period not specified, prospective cohort study	Adults, *n* = 70, all BSI, all HIV patients	Ciprofloxacin PO 1 g/day, in 2 doses/day 10 days (started after NTS isolation from blood culture)	Total case fatality after 1 month: 10/70 (14%) Recurrence: 63/70 (90%)
Epidemiological comparison between regimens			
Molyneux, 2009 [80], Malawi, 1997–2006, retrospective cohort study	Children, *n* = 105, all meningitis HIV positivity in 49/105 (47%)	**1997–2001**: Chloramphenicol IV 100 mg/kg/day in 4 doses/day for 14 days in 21/29 Ceftriaxone IV 100 mg/kg/day in 2 doses/day for 10 days in 8/29 **2002–2006**: Ceftriaxone IV 100 mg/kg/day in 2 doses/day for 10 days (76/76) + ciprofloxacin PO 20 mg/kg/day in 2 doses/day for 14 days (76/76)	In-hospital case fatality: 1997–2001: 14/29 (48.2%) 2002–2006: 41/76 (53.9%) Post-discharge case fatality: 1997–2001: 3/29 (10.3%) 2002–2006: 4/76 (5.3%) Recurrences: 1997–2001: 9/15 (60%), from which 7 HIV+ 2002–2006: 3/35 (8.6%), from which 1 HIV+ Sequelae: 1997–2001: 11/12 (91.7%) 2002–2006: 13/31 (41.9%)
Antimicrobial treatment duration			
Tsai, 2007 [87], Taiwan, 1996–2003, retrospective cohort study	Children, *n* = 184, all BSI	**Group < 7 days**: Median 5 days antimicrobial treatment Ceftriaxone/cefotaxime in 31/49, ampicillin in 16/49, other in 2/49 **Group ≥ 7 days**: Median 9.5 days antimicrobial treatment Ceftriaxone/cefotaxime in 121/135, ampicillin in 14/135	In-hospital case fatality: 0/184 (0%) Post-discharge case fatality: 0/184 (0%) Recurrences: 0/184 (0%) Persistent bacteremia: Group < 7 days: 1/21 (5%) Group ≥ 7 days: 1/43 (2%)
Hess, 2019 [88], USA, 2007–2016, retrospective cohort study	Children, *n* = 51, all BSI	Initial treatment: Ceftriaxone/cefotaxime IV in 48/51, others in 3/51 **Group < 7 days IV treatment**: Median 4 days IV treatment Switch to amoxicillin, cotrimoxazole, third-generation cephalosporins or ciprofloxacin after < 7 days **Group ≥ 7 days IV treatment**: Median 9 days IV treatment Switch to amoxicillin, cotrimoxazole, third-generation cephalosporins or ciprofloxacin after ≥ 7 days or 10 days ceftriaxone/cefotaxime IV	Recurrences: 30-day readmission / emergency visit: Group < 7 days IV treatment: 0/32 (0%) Group ≥ 7 days IV treatment: 1/19 (5%) Persistent bacteremia: Group < 7 days IV treatment: 7/32 (53%) Group ≥ 7 days IV treatment: 9/19 (47%)

*BSI* bloodstream infection, *PO* per os, *IV* intravenous, *h* hours

*One study was a study on a subgroup [77] from another included study [32]. As data on fever clearance were only reported in the subgroup study [77], the fever clearance data were taken into account and the other data from the subgroup study were disregarded

From the 36 publications with recommendations on antimicrobial treatment, six were national guidelines (Table 3) [97, 98, 108, 112, 115, 119]. The remaining 30 publications were expert opinion papers (*n* = 3) [105, 113, 114], narrative reviews (*n* = 26) [5, 6, 75, 89–96, 99–104, 109–112, 116–118, 120], and a systematic review [121]. One paper reported primary research data and formulated treatment recommendations after an extensive literature review [83]. Sixteen publications based their recommendations on observational or case studies, 16 publications referred to other reviews or guidelines, and four publications referred to the evidence available for enteric fever. Twelve papers with treatment recommendations did not refer to any published evidence in humans (Table 3). We appraised 21 publications as low quality and 15 as moderate quality (Additional File 3).

**Table 3:** Table summarizing the recommended antimicrobial treatment regimens to treat invasive non-typhoidal *Salmonella* infections

Antibiotic class	Antibiotic agent	Treatment	Recommended dosage and duration	Guideline (country, year)	Recommendation based on			
					Observational/case studies	Other reviews/guidelines	Evidence in enteric fever	No reference to literature*
Penicillins	Ampicillin, amoxicillin	First or second choice (if susceptible)	**Dose:** Children: 90–200 mg/kg/day in 3 doses/day Adults: 10–12 g/day		[89]	[91–93]		[96]
Trimethoprim-sulfamethoxazole	Trimethoprim-sulfamethoxazole	First or second choice (if susceptible)	**Dose:** Children: 40/8 mg/kg/day in 2 doses/day Adults: 1600/320 mg/day in 2 doses/day or 24/4.8–30/6 mg/kg/day in 3 doses/day	US, 2019 [97] US, 2018 [98]	[91, 99, 100]	[91, 92, 94, 95, 101]		[93, 96–98, 102]
Chloramphenicol	Chloramphenicol	First or second choice (if susceptible)	**Dose:** Children and adults: 50–100 mg/kg/day		[75, 89, 100, 103]	[93, 100]		[92, 102]
Third-generation cephalosporins					[75, 90, 99, 103, 104]	[94, 95, 104]		[105]
	Ceftriaxone	First or second choice	**Dose:** Children: 50–100 mg/kg/day in 1–2 doses/day Adults: or 75 mg/kg/day or 1–2 g/day in 1 dose/day	US, 2018 [98] US, 2019 [97] US, 2018 [106] CA, 2019 [107] FR, 2017 [108]	[89, 91, 100, 109, 110]	[91, 93, 100–102, 108–111]	[5, 92]	[6, 96–98, 106, 112]
	Cefotaxime	First or second choice	**Dose:** Children: 100–200 mg/kg/day in 4 doses/day Adults: 1–6 g/day in 3–6 doses/day	US, 2019 [97]	[83, 89, 91, 100, 110, 113]	[91, 93, 100, 101] [110] [111]		[97, 112]
	Ceftazidime, cefoperazone, moxalactam, aztreonam	Second choice			[83]	[93, 114]	[92]	
Fluoroquinolones				US, 2018 [106] FR, 2008 [115]	[6, 90, 99, 100, 104, 116]	[94, 95, 102, 104]	[90]	[96, 105, 106, 117]
	Ciprofloxacin	First or second choice	**Dose:** Children: 20 mg/kg/day IV or 20–30 mg/kg/day PO in 2 doses/day Adults: 800 mg IV or 1000–1500 mg PO in 2 doses/day	US, 2019 [97] US, 2018 [98]	[5, 75, 89, 92, 109, 110]	[93, 98, 101, 108–110]	[115]	[97, 98, 118]
	Gatifloxacin	Second choice	**Dose:** 10 mg/kg/day				[5]	
	Levofloxacin, moxifloxacin	Second choice	**Dose:** Adults: levofloxacin: 750 mg/day PO/IV in 1 dose/day Adults: moxifloxacin: 400 mg/day PO/IV in 1 dose/day	US, 2019 [97] US, 2018 [98]		[101]		[98]
	Pefloxacin, ofloxacin, norfloxacin, trovafloxacin	Second choice			[110]	[93]		
Macrolides	Azithromycin	Second choice	**Dose:** Children: 10–20 mg/kg/day PO	CA, 2019 [107] US, 2018 [106] US, 2018 [98]	[91]	[91, 102, 107]	[5]	[6, 98, 106]
Carbapenems	Imipenem, ertapenem, meropenem	Second choice			[110]		[5]	[91, 102]
Tigecycline	Tigecycline	Second choice					[5]	[91]

*PO* per os, *IV* intravenous, *US* United States, *CA* Canada, *FR* France

*Or only reference to conference proceedings, studies on *Salmonella* enteritis or in vitro/in vivo experimental studies

### Evidence on antimicrobial treatment efficacy and safety

Table 2 gives a detailed overview of the population, antimicrobial treatment regimens, and outcomes per included study. Large differences in study population and setting might affect the observed treatment outcome, e.g., the in-hospital case fatality for intravenous chloramphenicol treatment was higher in children with meningitis (49%) [43] than in children with bloodstream infection (24%) [81] and higher in cohorts of patients with HIV [76]. In HIV, high recurrence rates (43%) were seen after 14 days of chloramphenicol [76]. In 85% of patients with HIV and bloodstream infection, fever resolved within 72 h with chloramphenicol [74].

With C3G, fever often resolved within 3 days [77, 83]. In Rwandan children from whom 220/246 had a bloodstream infection, cefotaxime treatment resulted in an in-hospital case fatality of 11%, compared to 74% in children who did not receive cefotaxime. However, the latter group included children that died before the blood culture results were available, which introduced a large bias in the study results [32]. Recurrence rates were low after C3G treatment (Table 2) [83, 84]. However, a high proportion of persistent bacteremia (4/11) was seen in cefamandole regimens, a first-generation cephalosporin, despite in vitro susceptibility [82].

In fluoroquinolone-based treatment regimens, case fatality ratios varied between 3 and 14% (Table 2). Recurrences were often seen in patients with HIV [79, 86] but did not occur in children from DR Congo [78]. In the latter study, microbiological clearance occurred within 48–72 h after the start of oral ciprofloxacin treatment in all children, and possible fluoroquinolone side effects were observed in 3/36 children, i.e., one child with sickle cell anemia had increased bilirubin measurements, one child had raised aminotransferase enzymes that resolved upon the termination of ciprofloxacin, and one child had a swollen knee that resolved within 48 h. In none of them, ciprofloxacin treatment was interrupted earlier [78].

A study in Malawian children with meningitis compared the clinical outcome of two different treatment regimens epidemiologically. During the 5 years that ceftriaxone followed by fluoroquinolones was the standard treatment, the in-hospital case fatality was similar, but less fatal cases after discharge, less recurrences, and less sequelae occurred (Table 2) [80]. In children with bloodstream infection, a study in Taiwan compared a total antimicrobial treatment duration of < 7 days with one of ≥ 7 days [87], and a study in the USA compared < 7 days intravenous antimicrobial treatment with ≥ 7 days of intravenous antimicrobials [88]. They reported similar outcomes in both groups with the exception of slightly higher proportions of persistent bacteremia in the < 7 days groups (Table 2). It was however not clear at which treatment day the repeat blood cultures were taken.

#### Recommendations on antimicrobial treatment of invasive NTS infections

To treat invasive NTS infections, both the “Access group” antibiotics ampicillin, trimethoprim-sulfamethoxazole, and chloramphenicol, and C3G (ceftriaxone or cefotaxime) or fluoroquinolones (ciprofloxacin) from the “Watch group” antibiotics were often recommended as the first-choice antibiotics (Table 3). Some of these antibiotics were sporadically considered as the second choice due to the high prevalence of resistance, intolerance, safety concerns, or lack of treatment efficacy data (Additional File 2).

Some authors had concerns about the use of chloramphenicol to treat invasive NTS infections due to a possible lack of bactericidal activity [92, 99], high relapse rates [92, 93], no effect on carrier states [92, 93], and the need for long treatment courses [93]. In focal infections (osteomyelitis and endovascular infections), clinical failure was mentioned [89, 100]. In NTS meningitis, poor clinical efficacy and prolonged culture positivity of the cerebrospinal fluid were mentioned [99, 122]. Finally, some authors cited chloramphenicol safety issues, i.e., bone marrow depression and hepatic toxicity [92, 93, 99].

The need for clinical efficacy data and clinical trials on fluoroquinolones and C3G was already stated in 1986 and 1988 [95, 122]. For fluoroquinolones, the most frequent adverse events would be mild to moderate, reversible, gastrointestinal, dermatological, or neurological disturbances. The potential joint toxicity or cartilage destruction, particularly in children, was based on animal studies, from which the relevance in humans remains unclear [99, 116]. In addition, authors warranted that the use of fluoroquinolones to treat NTS could promote resistance in tuberculosis [6]. Thirdly, fluoroquinolones should not be used when resistance to nalidixic acid or decreased ciprofloxacin susceptibility is observed due to the risk of treatment failure [98, 109, 114, 115]. Finally, contradictory statements were made on the efficacy of fluoroquinolones in meningitis [91, 110] and the added value of combining C3G with fluoroquinolones (Additional File 2) [90, 91, 114].

Fluoroquinolones other than ciprofloxacin and C3G other than ceftriaxone and cefotaxime were generally listed as second-choice antimicrobial treatment options (Table 3). The use of azithromycin as a second-choice antibiotic was recommended in various publications, including the Red Book, the Sanford Guide to Antimicrobial Therapy, and the Canadian National Guidelines (Table 3). The need for studies on azithromycin use in NTS infections was explicitly stated in 2004 [114].

The recommendation for the duration of antimicrobial treatment differed according to the site of infection. For bloodstream infections, oral or intravenous antibiotic courses of 7–14 days were generally recommended [5, 89–91, 94, 97, 98, 101, 104, 106, 110–115, 121], although for azithromycin, 5–7 days would be sufficient [5, 91, 107]. For bloodstream infections in HIV patients with < 200 CD4 cells/mm^2^, 2–6 weeks of antibiotics were recommended [97, 109, 123]. For meningitis, 3–6 weeks antibiotic courses were recommended [89, 91, 99, 100, 103, 106, 109–111]. For osteomyelitis and other focal infections, 4–6 weeks of antibiotics were generally recommended [89, 91, 95, 106, 111, 112], although 2–4 weeks of fluoroquinolones or C3G were also suggested [114]. Finally, for (possible) endovascular infections, antibiotic courses of 4–6 weeks or even longer were recommended [90, 94, 95, 98, 114].

The use of oral fluoroquinolones to treat invasive NTS infections was recommended in various publications, including the Red Book, the Sanford Guide, and the NIH guidelines on opportunistic infections in patients with AIDS [97, 98, 106, 108, 110, 116]. The Red Book suggested that to treat bloodstream infections, intravenous ceftriaxone can be switched to oral fluoroquinolones or azithromycin when the blood culture is cleared and focal infections are excluded [106]. Sequential intravenous and oral treatment in children over 12 months with bloodstream infection was also recommended in a review [91]. In the treatment of a first bloodstream infection in patients with AIDS, 1–2 weeks of intravenous antibiotics could be continued for 4 weeks with an oral fluoroquinolone [114]. To treat osteomyelitis, intravenous antibiotics could be switched to oral antibiotics if systemic symptoms resolved [110].

## Discussion

### Summary of findings

This systematic review and meta-analysis provides a spatiotemporal overview of AMR in invasive NTS infections in sub-Saharan Africa and an extensive overview of all available evidence and recommendations on its antimicrobial treatment. Since 2001, MDR was observed in more than half of the NTS isolates in almost all sub-Saharan African countries. In addition, we report the presence of C3G resistance and FQNS in all four sub-Saharan African regions. The meta-analysis revealed an increase in the pooled proportion of C3G resistance up to 5% resistant isolates after 2010. The emergence of FQNS was more variable and was not reflected in increasing pooled proportions over time. Nevertheless, the presence of > 5% FQNS was reported in South Africa [56, 58], Ethiopia [61], Kenya [60], Mozambique [40, 67], Ghana [41, 68], and Burkina Faso [37]. Although no official breakpoints on azithromycin susceptibility exist yet, the presence of azithromycin-resistant NTS was suggested in DR Congo. Full carbapenem susceptibility was observed in the few studies that reported on carbapenems. Susceptibility data for first- or second-generation cephalosporins or aminoglycosides were often reported. These antibiotics are included in routine antibiotic susceptibility testing panels for *Enterobacterales*, and in the daily practice of a diagnostic laboratory, they are inoculated together with the identification test, i.e., before the identification of *Salmonella*. However, CLSI warns not to report *Salmonella* as susceptible to these antibiotics due to in vivo inefficacy [124]. Likewise, tetracycline susceptibility was often reported. Tetracycline is not included in the routine antibiotic susceptibility testing panel for Enterobacterales and is clinically irrelevant [125, 126], but is monitored for epidemiological purposes, in particular within the scope of foodborne and zoonotic salmonellosis [127, 128].

As expected, this review did not find high-quality evidence on the efficacy or safety of antimicrobial treatment of invasive NTS infections. No data were available from interventional or dedicated observational clinical studies. In analogy with the lack of evidence, we did not find supranational guidelines on the antimicrobial treatment of invasive NTS infections. However, we found three guidelines from the USA that are used internationally, i.e., the Red Book, the Sanford Guide to Antimicrobial Therapy, and the NIH guidelines on the prevention and treatment of opportunistic infections in patients with AIDS [97, 98, 106]. In general, the “Access group” antibiotics ampicillin, trimethoprim-sulfamethoxazole, and chloramphenicol and the “Watch group” antibiotics ceftriaxone, cefotaxime, and ciprofloxacin were recommended as the first-choice antibiotics. Some also recommended the use of the “Watch group” antibiotic azithromycin [7]. Most authors proposed 7–14 days of antibiotics for uncomplicated bloodstream infections and 4–6 weeks for meningitis, osteomyelitis, endovascular, or other focal infections. The possibility of oral antimicrobial treatment or switch to oral antimicrobial treatment was suggested in some guidelines and reviews.

### Limitations

This review had some limitations. Firstly, we had to exclude studies that did not report separate data for *Salmonella* (Para) Typhi and NTS or invasive and intestinal infections. As such, we may have missed some data on AMR and treatment. We could include only four studies that reported susceptibility data from NTS isolated before 1991, and from some sub-Saharan African countries, we could not include a single study. In addition, the temporal resolution was affected by compiled antibiotic susceptibility data from studies that reported in long time periods. Moreover, only nine from the 16 treatment efficacy studies reported data from sub-Saharan Africa. As the *Salmonella* Typhimurium and Enteritidis strains circulating in sub-Saharan Africa have a more invasive genotype and phenotype, data from NTS causing invasive infections in high-risk patients or transient bacteremia in other settings may not be generalizable to invasive NTS infections in sub-Saharan Africa.

Secondly, it is likely that publication and reporting bias favoring studies that reported resistant NTS occurred, particularly during the early years. However, MDR and FQNS will also have been underreported. The definition of MDR is a recent convention [5, 23, 24], and the first study that reported MDR was only published in 2012. Before the revised breakpoints in 2012, only FQR was detected [18, 19, 25], but nalidixic acid resistance suggested the presence of DCS in six studies [32, 38, 48, 61, 65, 72]. Even after the revision of the fluoroquinolone breakpoints, some studies did not clarify if DCS was taken into account while assessing FQNS.

Finally, a careful interpretation of the pooled proportions of MDR, C3G resistance, and FQNS is warranted. The heterogeneity was high in all meta-analyses. Although subgroup analysis per time period reduced the heterogeneity, the residual heterogeneity remained high. By meta-regression, we aimed to further reduce the heterogeneity, but we could not identify other study characteristics as effect moderators. It is however plausible that the study designs differed in too many aspects to identify the effect of specific characteristics. In addition, we decided to exclude the data from studies reporting susceptibility data for < 50 NTS, although these studies might still be representative for their study population, and when these studies were included in the meta-analysis, a trend in increasing FQNS was observed. Finally, we excluded studies with a grade E quality from the meta-analyses due to uncertainty or inconsistency in some of their reported data, while (most of) their data might be correct. By contrast, we included studies with a grade D quality in the meta-analysis, while the quality of these data cannot be fully assured neither.

### Comparison to other *Salmonella* infections

This study is the first systematic review and meta-analysis on AMR and antimicrobial therapy in invasive NTS infections. While recently two systematic reviews have been published on the emergence of AMR in enteric fever [129, 130], this systematic review and meta-analysis finally provides data on the spatiotemporal distribution of AMR in invasive NTS infections across sub-Saharan Africa. The pooled proportion of MDR in NTS was comparable with the proportion of MDR in *Salmonella* Typhi in sub-Saharan Africa in these two reviews [129, 130]. However, we observed more C3G resistance in NTS than in their data on *Salmonella* Typhi, while they reported more FQNS than our observations in NTS [129, 130]. Another recent systematic review and meta-analysis assessed the presence of MDR and FQNS in *Salmonella* Typhi and non-typhi infections in sub-Saharan Africa, without differentiating between intestinal and invasive infections. They reported similar pooled MDR and FQNS proportions. They also reported more MDR in *Salmonella* Typhimurium than in *Salmonella* Enteritidis, but reported more FQNS in *Salmonella* Enteritidis than in *Salmonella* Typhimurium [131]. Finally, it is remarkable that treatment efficacy data and evidence-based supranational guidelines are widely available for intestinal *Salmonella* infections and enteric fever [5, 123, 132–135], but are missing for invasive NTS infections which, in sub-Saharan Africa, have the highest disease burden of all three diseases [1, 2].

### Relevance and future implications

The widespread presence of MDR and subsequent emergence of C3G resistance and FQNS in invasive NTS infections in sub-Saharan Africa seriously challenge treatment. Due to the high burden of invasive NTS infections in sub-Saharan Africa [2] and the frequency of NTS as the cause of invasive infections [4], this treatment challenge provokes a considerable public health threat. Firstly, the current empiric antimicrobial treatment guidelines for possible severe bacterial infections in African children do not cover invasive MDR NTS infections [3, 133]. Secondly, to allow treatment with any of the current first-choice antibiotic options for invasive NTS infections, the emergence of C3G resistance and FQNS must be contained. Therefore, the global action plan against antimicrobial resistance states the importance of consistent collection and monitoring of surveillance data to strengthen our knowledge [10]. In the current context of emerging C3G resistance and FQNS, clinicians will sometimes have to rely on second-choice antibiotics. Interpretative criteria for azithromycin susceptibility testing in NTS isolates from invasive infections are therefore urgently needed. In addition, susceptibility data on second-choice antibiotics like azithromycin and carbapenems should be tested and included in surveillance reports. In low-resource settings, ciprofloxacin MIC testing is technically challenging and pefloxacin and nalidixic acid disk tests are more feasible alternatives to test fluoroquinolone susceptibility [136]. Therefore, surveillance studies should report results from ciprofloxacin MIC testing and surrogate pefloxacin or nalidixic acid disk tests together. As such, the performance of pefloxacin and nalidixic acid surrogate disk tests in sub-Saharan Africa can be assessed. Moreover, in the context of emerging FQNS, it may be useful to test and report susceptibility to gatifloxacin, which can be an effective alternative to treat DCS NTS, as it is less affected by a common FQNS molecular mechanism (*gyrA* mutation). Gatifloxacin has been withdrawn from the market in many countries due to dysglycemia in elderly and diabetic patients, but this patient profile is uncommon in invasive NTS infections in sub-Saharan Africa [5]. Finally, the reporting of combined resistance patterns, e.g., MDR combined with C3G resistance and azithromycin resistance, should be promoted by the use of convened acronyms and definitions of extensive drug resistance (XDR) and pan drug resistance (PDR), as recently proposed by our research group [137]. These combined resistance patterns provide information about the antibiotic options left for treatment. The use of internationally convened definitions and acronyms will harmonize reporting and facilitate advocacy and policymaking.

Secondly, the global action plan against antimicrobial resistance highlights the need to optimize the use of antibiotics [10]. However, the current scarcity of data on antimicrobial treatment of invasive NTS infections hampers antibiotic stewardship in sub-Saharan Africa, where NTS are situated in the top three of pathogens causing bloodstream infections [4]. To formulate evidence-based supranational treatment guidelines for invasive NTS infections, interventional studies and dedicated observational studies must urgently be organized to provide data on treatment efficacy and safety. To align with the WHO essential medicines list, with the current antibiotic recommendations and the current clinical antibiotic use in sub-Saharan Africa, studies must initially focus on C3G, fluoroquinolones, and azithromycin. In addition, special attention must be given to an early switch to oral antibiotics. In general, the evidence supporting the superiority of intravenous antibiotic courses for invasive infections is weak. Moreover, early switch to oral antibiotics will shorten the hospital stay, reduce costs, and reduce healthcare-associated infections, which are of particular importance in a low-resource setting [121, 138–140].

Thirdly, and again in line with the global action plan against antimicrobial resistance, the emerging resistance in and the lack of data on the optimal treatment of invasive NTS infections highlight the need for preventive measures to decrease the disease incidence [10]. Currently, vaccines against NTS that cause invasive infections are being developed [141]. None of the treatment efficacy studies in this review assessed the impact of antibiotic treatment on the duration of fecal carriage, although it is known that antibiotics prolong fecal shedding in NTS gastroenteritis. In invasive NTS infections in sub-Saharan Africa, the role of fecal carriage has not been fully elucidated yet. However, increasing evidence supports a human instead of zoonotic transmission of invasive NTS in sub-Saharan Africa [63, 142] (Phoba M-F, Barbé B, Ley B, Van Puyvelde S, Post A, Mattheus W, et al.: High genetic similarity between non-typhoidal Salmonella isolated from paired blood and stool samples of children in the Democratic Republic of the Congo, accepted for publication). To design the appropriate public health interventions, more research that clarifies the transmission of the NTS strains circulating in sub-Saharan Africa is needed.

## Conclusion

In this systematic review and meta-analysis, we demonstrated the widespread presence of MDR and the emergence of C3G resistance and FQNS in invasive NTS infections in all sub-Saharan African regions. In addition, we highlighted the lack of data on the efficacy and safety of antimicrobial treatment of these infections, which was reflected in the absence of supranational evidence-based guidelines. To contain the emergence of AMR in invasive NTS infections, close monitoring and harmonized reporting of surveillance data, including data on the presence of combined resistance patterns, are essential. Some of the current recommendations list azithromycin as a second-choice antibiotic to treat invasive NTS infections. However, to allow its rational use, interpretative criteria for its susceptibility testing are needed. Lastly, clinical studies must be organized to provide the much needed data on the treatment efficacy of different antibiotic regimens, including regimens with early switch to oral administration. These data will finally allow the development of evidence-based and internationally harmonized guidelines and facilitate antibiotic stewardship.

## Supplementary information

**Additional file 1: Table S1.** Prisma checklist. **List S2.** Search strategy used for MEDLINE and Ovid Embase database searches. **Table S3.** In- and exclusion criteria for both research questions. **List S4.** Published articles from which no full text was found. **Figure S5.** Sensitivity analysis for multidrug resistance, third generation cephalosporins & fluoroquinolone non-susceptibility. **Figure S6.** Meta-analysis and forest plots of multidrug resistance, third generation cephalosporin resistance and fluoroquinolone non-susceptibility according to NTS serotype. **Table S7.** Meta-regression to identify moderators of the pooled proportions of multidrug resistance (MDR), third generation cephalosporin resistance (C3G-resistance) and fluoroquinolone non-susceptibility (FQNS).

**Additional file 2.** Data extraction database and datasets for meta-analysis.

**Additional file 3.** Dataset from risk of bias assessment.

## Data Availability

The datasets supporting the conclusions of this article are included within the additional files of this article, i.e., Additional Files 2 and 3.

## Abbreviations

AMR: Antimicrobial resistance
C3G: Third-generation cephalosporins
DCS: Decreased ciprofloxacin susceptibility
FQNS: Fluoroquinolone non-susceptibility
FQR: Fluoroquinolone resistance
HIV: Human immunodeficiency virus
MDR: Multidrug resistance
NTS: Non-typhoidal *Salmonella*
WHO: World Health Organization
