# Supplementary material for: Invasive non-typhoidal Salmonella infections in sub-Saharan Africa: a systematic review on antimicrobial resistance and treatment
Source: BMC Med. 2020 Jul 17;18:212. doi: 10.1186/s12916-020-01652-4 (PMC7367361; doi:10.1186/s12916-020-01652-4)
Supplement: Supplementary file 1 — Additional file 1: Table S1. Prisma checklist. List S2. Search strategy used for MEDLINE and Ovid Embase database searches. Table S3. In- and exclusion criteria for both research questions. List S4. Published articles from which no full text was found. Figure S5. Sensitivity analysis for multidrug resistance, third generation cephalosporins & fluoroquinolone non-susceptibility. Figure S6. Meta-analysis and forest plots of multidrug resistance, third generation cephalosporin resistance and fluoroquinolone non-susceptibility according to NTS serotype. Table S7. Meta-regression to identify moderators of the pooled proportions of multidrug resistance (MDR), third generation cephalosporin resistance (C3G-resistance) and fluoroquinolone non-susceptibility (FQNS). [file 12916_2020_1652_MOESM1_ESM.docx]

# Supplemental materials

## Table S1. Prisma checklist

| **Section/topic** | **#** | **Checklist item** | **Reported on page** |
| --- | --- | --- | --- |
| **TITLE** | | |  |
| Title | 1 | Identify the report as a systematic review, meta-analysis, or both. | In title |
| **ABSTRACT** | | |  |
| Structured summary | 2 | Provide a structured summary including, as applicable: background; objectives; data sources; study eligibility criteria, participants, and interventions; study appraisal and synthesis methods; results; limitations; conclusions and implications of key findings; systematic review registration number. | Page 2 - 3 |
| **INTRODUCTION** | | |  |
| Rationale | 3 | Describe the rationale for the review in the context of what is already known. | Page 3 - 4 |
| Objectives | 4 | Provide an explicit statement of questions being addressed with reference to participants, interventions, comparisons, outcomes, and study design (PICOS). | Page 4 |
| **METHODS** | | |  |
| Protocol and registration | 5 | Indicate if a review protocol exists, if and where it can be accessed (e.g., Web address), and, if available, provide registration information including registration number. | Page 5 |
| Eligibility criteria | 6 | Specify study characteristics (e.g., PICOS, length of follow-up) and report characteristics (e.g., years considered, language, publication status) used as criteria for eligibility, giving rationale. | Page 5 - 6 + Additional file 1: Table S3 |
| Information sources | 7 | Describe all information sources (e.g., databases with dates of coverage, contact with study authors to identify additional studies) in the search and date last searched. | Page 5 |
| Search | 8 | Present full electronic search strategy for at least one database, including any limits used, such that it could be repeated. | Additional file 1: List S2 |
| Study selection | 9 | State the process for selecting studies (i.e., screening, eligibility, included in systematic review, and, if applicable, included in the meta-analysis). | Page 5 |
| Data collection process | 10 | Describe method of data extraction from reports (e.g., piloted forms, independently, in duplicate) and any processes for obtaining and confirming data from investigators. | Page 5 - 6 |
| Data items | 11 | List and define all variables for which data were sought (e.g., PICOS, funding sources) and any assumptions and simplifications made. | Page 5 - 6 and Additional file 2 |
| Risk of bias in individual studies | 12 | Describe methods used for assessing risk of bias of individual studies (including specification of whether this was done at the study or outcome level), and how this information is to be used in any data synthesis. | Page 6 |
| Summary measures | 13 | State the principal summary measures (e.g., risk ratio, difference in means). | Page 6 |
| Synthesis of results | 14 | Describe the methods of handling data and combining results of studies, if done, including measures of consistency (e.g., I^2^) for each meta-analysis. | Page 7 - 8 |

| **Section/topic** | **#** | **Checklist item** | **Reported on page** |
| --- | --- | --- | --- |
| Risk of bias across studies | 15 | Specify any assessment of risk of bias that may affect the cumulative evidence (e.g., publication bias, selective reporting within studies). | Page 6 - 8 |
| Additional analyses | 16 | Describe methods of additional analyses (e.g., sensitivity or subgroup analyses, meta-regression), if done, indicating which were pre-specified. | Page 7 - 8 |
| **RESULTS** | | |  |
| Study selection | 17 | Give numbers of studies screened, assessed for eligibility, and included in the review, with reasons for exclusions at each stage, ideally with a flow diagram. | Page 8 - 9 + page 14 |
| Study characteristics | 18 | For each study, present characteristics for which data were extracted (e.g., study size, PICOS, follow-up period) and provide the citations. | Page 8 - 9 + page 14 |
| Risk of bias within studies | 19 | Present data on risk of bias of each study and, if available, any outcome level assessment (see item 12). | Additional file 3 |
| Results of individual studies | 20 | For all outcomes considered (benefits or harms), present, for each study: (a) simple summary data for each intervention group (b) effect estimates and confidence intervals, ideally with a forest plot. | Page 10 - 18 |
| Synthesis of results | 21 | Present results of each meta-analysis done, including confidence intervals and measures of consistency. | Page 10 - 13 |
| Risk of bias across studies | 22 | Present results of any assessment of risk of bias across studies (see Item 15). | Page 10 – 13 |
| Additional analysis | 23 | Give results of additional analyses, if done (e.g., sensitivity or subgroup analyses, meta-regression [see Item 16]). | Page 10 – 13 + Additional file 1: S5 – S8 |
| **DISCUSSION** | | |  |
| Summary of evidence | 24 | Summarize the main findings including the strength of evidence for each main outcome; consider their relevance to key groups (e.g., healthcare providers, users, and policy makers). | Page 18 - 19 |
| Limitations | 25 | Discuss limitations at study and outcome level (e.g., risk of bias), and at review-level (e.g., incomplete retrieval of identified research, reporting bias). | Page 19 - 20 |
| Conclusions | 26 | Provide a general interpretation of the results in the context of other evidence, and implications for future research. | Page 21 - 23 |
| **FUNDING** | | |  |
| Funding | 27 | Describe sources of funding for the systematic review and other support (e.g., supply of data); role of funders for the systematic review. | Page 23 |

*From:*  Moher D, Liberati A, Tetzlaff J, Altman DG, The PRISMA Group (2009). Preferred Reporting Items for Systematic Reviews and Meta-Analyses: The PRISMA Statement. PLoS Med 6(7): e1000097. doi:10.1371/journal.pmed1000097

For more information, visit: **www.prisma-statement.org**.

Page 2 of 2

## List S1. Search strategy used for MEDLINE and Ovid Embase database searches

### MEDLINE - Research question 1: Antimicrobial resistance in invasive NTS infections in Sub-Saharan Africa

**CONCEPT: Antimicrobial resistance**

((((“Drug Resistance"[Mesh:NoExp] OR Drug-Resistan*[Tiab] OR "Drug Resistance, Microbial"[Mesh] OR "Microbial Drug Resistance”[Tiab] OR “Microbial Drug Resistant”[Tiab] OR "Drug Resistance, Bacterial"[Mesh:NoExp] OR Bacterial-resistan*[Tiab] OR “Bacterial Drug Resistance”[Tiab] OR “Bacterial Drug Resistant”[Tiab] OR Antibiotic-resistan*[Tiab] OR “Antibiotic susceptibility”[Tiab] OR “Antibiotic sensitivity”[Tiab] OR Antimicrobial-resistan*[Tiab] OR “antimicrobial drug resistance”[Tiab] OR “antimicrobial drug resistant”[Tiab] OR “Antimicrobial susceptibility”[Tiab] OR “Antimicrobial sensitivity”[Tiab] OR “antimicrobial drug sensitivity”[Tiab] OR “antimicrobial drug suspectibility”[Tiab] OR resistan*[Tiab] OR susceptib*[Tiab] OR sensitiv*[Tiab] OR subsensivit*[Tiab] OR “antibacterial drug resistance”[Tiab] OR “antibacterial drug resistant”[Tiab] OR antibacterial-resistan*[Tiab] OR “antibiotic non-suspectibility”[Tiab] OR Multidrug-resistan*[Tiab] OR “Multi-drug resistance”[Tiab] OR “Multi-drug resistant”[Tiab] OR MDR[Tiab] OR “multiple drug resistance”[Tiab] OR “multiple drug resistant”[Tiab] OR "Drug Resistance, Multiple, Bacterial"[Mesh] OR “beta lactam”[Tiab] OR “betalactam”[Tiab] OR “b-lactam”[Tiab] OR blactam*[Tiab] OR "Beta-Lactam Resistance"[Mesh] OR "beta-Lactamases"[Mesh] OR “beta-lactamase”[Tiab] OR “betalactamase”[Tiab] OR ESBL[Tiab] OR "Penicillins"[Mesh] OR Penicillin*[Tiab] OR PEN-Resistan*[Tiab] OR "Ampicillin"[Mesh] OR Ampicillin[Tiab] OR Amp-Resistan*[Tiab] OR "Amoxicillin"[Mesh] OR Amoxicillin*[Tiab] OR "Trimethoprim"[Mesh] OR "Trimethoprim Resistance"[Mesh] OR Trimethoprim*[Tiab] OR TMP-Resistan*[Tiab] OR "Sulfamethoxazole"[Mesh] OR Sulfamethoxazol*[Tiab] OR Cotrimoxazol*[Tiab] OR “Co-Trimoxazole” [Tiab] OR “Co-Trimoxazol” [Tiab] OR “Trimethoprim-Sulfamethoxazole”[Tiab] OR “Trimethoprim-Sulfamethoxazol”[Tiab] OR “Sulfamethoxazole-Trimethoprim”[Tiab] OR “Sulfamethoxazol-Trimethoprim”[Tiab] OR “sulfamethoxazole and trimethoprim”[Tiab] OR “sulfamethoxazole plus trimethoprim”[Tiab] OR “trimethoprim plus sulfamethoxazole”[Tiab] OR “trimethoprim sulfamethoxazole combination”[Tiab] OR “trimethoprimsulfamethoxazole”[Tiab] OR “trimetoprim-sulfa”[Tiab] OR “trimetoprimsulfamethoxazole”[Tiab] OR "Chloramphenicol"[Mesh] OR "Chloramphenicol Resistance"[Mesh] OR Chloramphenicol[Tiab] OR CA-Resistan*[Tiab] OR "Cephalosporins"[Mesh] OR Cephalosporin*[Tiab] OR Cefalosporin*[Tiab] OR "Ceftriaxone"[Mesh] OR Ceftriaxon*[Tiab] OR “Cefotaxime”[Mesh] OR Cefotaxim*[Tiab] OR CTX-Resistan*[Tiab] OR “Ceftazidime”[Mesh] OR Ceftazidim*[Tiab] OR CAZ-Resistan*[Tiab] OR "Cefixime"[Mesh] OR Cefixim*[Tiab] OR "Ciprofloxacin"[Mesh] OR Ciprofloxacin*[Tiab] OR CIP-resistan*[Tiab] OR “DCS”[Tiab] OR "Ofloxacin"[Mesh] OR “Ofloxacin"[Tiab] OR "Nalidixic Acid"[Mesh] OR "Nalidixic Acid"[Tiab] OR “pefloxacin”[Mesh] OR “pefloxacin”[Tiab] OR "Gatifloxacin"[Mesh] OR "Gatifloxacin"[Tiab] OR "Fluoroquinolones"[Mesh] OR fluoroquinolon*[Tiab] OR “fluorinated quinolone resistance”[Tiab] OR “fluorinated quinolone resistant”[Tiab] OR “fluoro-quinolone resistance”[Tiab] OR “fluoro-quinolone resistant”[Tiab] OR fluoroquinolones-resistan*[Tiab] OR “Azithromycin"[Mesh] OR azithromycin*[Tiab] OR "Macrolides"[Mesh] OR macrolide*[Tiab] OR "Tigecycline"[Mesh] OR "Tigecycline"[Tiab] OR "temocillin" [Supplementary Concept] OR "temocillin"[Tiab] OR "Meropenem"[Mesh] OR "Meropenem"[Tiab] OR "Carbapenems"[Mesh] OR "Carbapenems"[Tiab] OR "Colistin"[Mesh] OR "Colistin"[Tiab] OR "Aztreonam"[Mesh] OR "Aztreonam"[Tiab] OR "Sulfonamides"[Mesh] OR sulphonamide*[Tiab] OR sulfonamide*[Tiab] OR perfloxacin*[Tiab] OR "Levofloxacin"[Mesh] OR levofloxacin*[Tiab]))

**AND**

**CONCEPT: Non-typhoidal *Salmonella***

(((((("Salmonella enteritidis"[Mesh] OR "Salmonella enteritidis" [Tiab] OR "Salmonella typhimurium"[Mesh] OR "Salmonella typhimurium" [Tiab] OR "nontyphoidal salmonella"[Tiab] OR "non typhoidal salmonella"[Tiab] OR "non typhi salmonella"[Tiab] OR "Non typhoidal"[Tiab] OR "Nontyphoidal"[Tiab] OR "Non typhi"[Tiab] OR Salmonella-Infection*[Mesh] OR Salmonella-Infection* [Tiab] OR Salmonellosis [Tiab] OR Salmonelloses [Tiab] OR Salmonella[Mesh] OR Salmonella[Tiab])))

**AND**

**CONCEPT: Invasive infection**

(("Bacteremia"[Mesh] OR Bacteremia [Tiab] OR bacteraemia [Tiab] OR bacteriemia[Tiab] OR "Sepsis"[Mesh] OR sepsis [Tiab] OR “sepsis syndrome”[Tiab] OR “septic disease”[Tiab] OR Septicemia* [Tiab] OR Septicaemia* [Tiab] OR "Invasive"[Tiab] OR Extraintestinal-infection*[Tiab] OR "Extra intestinal infection"[Tiab] OR "Extra intestinal infections"[Tiab] OR septi*[Tiab] OR blood*[Tiab] OR bloodstream*[Tiab] OR bloodculture[Tiab] OR “blood-culture”[Tiab] OR [hemoculture])))

**AND**

**CONCEPT: sub-Saharan Africa**

(("Africa South of the Sahara"[Mesh] OR "sub-saharan Africa"[Tiab] OR "subsaharan Africa"[Tiab] OR "Sub Saharan Africa"[Tiab] OR "Africa, Central"[Mesh] OR "Central Africa"[Tiab] OR "Cameroon"[Mesh] OR "Cameroon"[Tiab] OR "Central African Republic"[Mesh] OR "Central African Republic"[Tiab] OR "Chad"[Mesh] OR "Chad"[Tiab] OR "Congo"[Mesh] OR "Congo"[Tiab] OR "Democratic Republic of the Congo"[Mesh] OR "Democratic Republic of the Congo"[Tiab] OR "Equatorial Guinea"[Mesh] OR "Equatorial Guinea"[Tiab] OR "Gabon"[Mesh] OR "Gabon"[Tiab] OR "Sao Tome and Principe"[Mesh] OR "Sao Tome and Principe"[Tiab] OR "Africa, Eastern"[Mesh] OR "Eastern Africa"[Tiab] OR "East Africa"[Tiab] OR "Burundi"[Mesh] OR "Burundi"[Tiab] OR "Djibouti"[Mesh] OR "Djibouti"[Tiab] OR "Eritrea"[Mesh] OR "Eritrea"[Tiab] OR "Ethiopia"[Mesh] OR "Ethiopia" [Tiab] OR "Kenya"[Mesh] OR "Kenya" [Tiab] OR "Rwanda"[Mesh] OR "Rwanda" [Tiab] OR "Somalia"[Mesh] OR "Somalia"[Tiab] OR "South Sudan"[Mesh] OR "South Sudan"[Tiab] OR "Sudan"[Mesh] OR "Sudan"[Tiab] OR "Tanzania"[Mesh] OR "Tanzania"[Tiab] OR "Uganda"[Mesh] OR "Uganda"[Tiab] OR "Africa, Southern"[Mesh] OR "Southern Africa"[Tiab] OR "South Africa"[Tiab] OR "Angola"[Mesh] OR "Angola"[Tiab] OR "Botswana"[Mesh] OR "Botswana"[Tiab] OR "Lesotho"[Mesh] OR "Lesotho"[Tiab] OR "Malawi"[Mesh] OR "Malawi"[Tiab] OR "Mozambique"[Mesh] OR "Mozambique"[Tiab] OR "Namibia"[Mesh] OR "Namibia"[Tiab] OR "South Africa"[Mesh] OR "South Africa"[Tiab] OR "Swaziland"[Mesh] OR "Swaziland"[Tiab] OR "Zambia"[Mesh] OR "Zambia"[Tiab] OR "Zimbabwe"[Mesh] OR "Zimbabwe"[Tiab] OR "Africa, Western"[Mesh] OR "Western Africa"[Tiab] OR "West Africa"[Tiab] OR "Benin"[Mesh] OR "Benin"[Tiab] OR "Burkina Faso"[Mesh] OR "Burkina Faso”[Tiab] OR "Cabo Verde"[Mesh] OR "Cabo Verde"[Tiab] OR "Cote d'Ivoire"[Mesh] OR "Cote d'Ivoire"[Tiab] OR "Gambia"[Mesh] OR "Gambia"[Tiab] OR "Ghana"[Mesh] OR "Ghana"[Tiab] OR "Guinea"[Mesh] OR "Guinea"[Tiab] OR "Guinea-Bissau"[Mesh] OR "Guinea-Bissau"[Tiab] OR "Liberia"[Mesh] OR "Liberia"[Tiab] OR "Mali"[Mesh] OR "Mali"[Tiab] OR "Mauritania"[Mesh] OR "Mauritania"[Tiab] OR "Niger"[Mesh] OR "Niger"[Tiab] OR "Nigeria"[Mesh] OR "Nigeria"[Tiab] OR "Senegal"[Mesh] OR "Senegal"[Tiab] OR "Sierra Leone"[Mesh] OR "Sierra Leone"[Tiab] OR "Togo"[Mesh] OR "Togo"[Tiab] OR "Comoros"[Mesh] OR "Comoros"[Tiab] OR “Mayotte”[Tiab] OR "Madagascar"[Mesh] OR "Madagascar"[Tiab] OR “Sahel”[Tiab]))))

### MEDLINE - Research question 2: Antimicrobial therapy for invasive NTS infections

### **CONCEPT: Antimicrobial treatment**

(((("Therapeutics"[Mesh:NoExp] OR "Drug Therapy"[Mesh] OR “Drug therapy”[Tiab] OR “Drug therapies”[Tiab] OR Drug-treatment*[Tiab] OR “Antibiotic therapy”[Tiab] OR “Antibiotic therapies”[Tiab] OR Antibiotic-treatment*[Tiab] OR “Antibiotic management”[Tiab] OR “Antibacterial therapy”[Tiab] OR “Antibacterial therapies”[Tiab] OR “Anti-bacterial therapy”[Tiab] OR “Anti-bacterial therapies”[Tiab] OR Antibacterial-treatment*[Tiab] OR “Anti-bacterial treatment”[Tiab] OR “Anti-bacterial treatments”[Tiab] OR "Anti-Infective Agents"[Mesh] OR “anti-infective agent”[Tiab] OR “anti-infective agents”[Tiab] OR antiinfective-agent*[Tiab] OR "Anti-Bacterial Agents "[Mesh] OR “Anti-Bacterial Agent”[Tiab] OR “Anti-Bacterial Agents”[Tiab] OR Antibacterial-Agent*[Tiab] OR “Anti-Bacterial Drug”[Tiab] OR “Anti-Bacterial Drugs”[Tiab] OR Antibacterial-Drug*[Tiab] OR Antimicrobial-Agent*[Tiab] OR Antimicrobial compound*[Tiab] OR Antimicrobial-Drug*[Tiab] OR Antiseptic-Agent*[Tiab] OR therapy[Tiab] OR therapies[Tiab] OR treatment*[Tiab] OR management[Tiab] OR therapeutic*[Tiab] OR antibiotic*[Tiab] OR antimicrobial[Tiab])))

**AND**

**CONCEPT: Non-typhoidal *Salmonella***

(((((("Salmonella enteritidis"[Mesh] OR "Salmonella enteritidis" [Tiab] OR "Salmonella typhimurium"[Mesh] OR "Salmonella typhimurium" [Tiab] OR "nontyphoidal salmonella"[Tiab] OR "non typhoidal salmonella"[Tiab] OR "non typhi salmonella"[Tiab] OR "Non typhoidal"[Tiab] OR "Nontyphoidal"[Tiab] OR "Non typhi"[Tiab] OR Salmonella-Infection*[Mesh] OR Salmonella-Infection* [Tiab] OR Salmonellosis [Tiab] OR Salmonelloses [Tiab] OR Salmonella[Mesh] OR Salmonella[Tiab])))

**AND**

**CONCEPT: Invasive infection**

(("Bacteremia"[Mesh] OR Bacteremia [Tiab] OR bacteraemia [Tiab] OR bacteriemia[Tiab] OR "Sepsis"[Mesh] OR sepsis [Tiab] OR “sepsis syndrome”[Tiab] OR “septic disease”[Tiab] OR Septicemia* [Tiab] OR Septicaemia* [Tiab] OR "Invasive"[Tiab] OR Extraintestinal-infection*[Tiab] OR "Extra intestinal infection"[Tiab] OR "Extra intestinal infections"[Tiab] OR septi*[Tiab] OR blood*[Tiab] OR bloodstream*[Tiab] OR bloodculture[Tiab] OR “blood-culture”[Tiab] OR [hemoculture])))

### Ovid embase - Research question 1: Antimicrobial resistance in invasive NTS infections in Sub-Saharan Africa

**CONCEPT: Antimicrobial resistance**

‘drug resistance’/exp OR ‘drug resistan*’:ti,ab,kw OR ‘microbial drug resistan*’:ti,ab,kw OR ‘bacterial resistan*’:ti,ab,kw OR ‘bacterial drug resistan*’:ti,ab,kw OR ‘bacterium resistan*’:ti,ab,kw OR ‘antibiotic resistance'/exp OR 'antibiotic resistan*':ti,ab,kw OR 'antibiotic sensitivity'/exp OR 'antibiotic sensitivity':ti,ab,kw OR ‘antibiotic susceptibility’:ti,ab,kw OR ‘antimicrobial drug resistan*’:ti,ab,kw OR ‘antimicrobial resistan*’:ti,ab,kw OR ’antimicrobial sensitivity’:ti,ab,kw OR ‘antimicrobial susceptibility’:ti,ab,kw OR ‘antimicrobial drug sensitivity’:ti,ab,kw OR ‘antimicrobial drug susceptibility’:ti,ab,kw OR ‘resistan*’:ti,ab,kw OR ‘susceptib*’:ti,ab,kw OR ‘sensitiv*’:ti,ab,kw OR ‘subsensitiv*’:ti,ab,kw OR ‘antibacterial drug resistan*’:ti,ab,kw OR ‘antibacterial resistan*’:ti,ab,kw OR ‘antibiotic non-susceptibility’:ti,ab,kw OR ‘antibiotic nonsusceptibility’:ti,ab,kw OR 'drug sensitivity'/de OR ‘multidrug resistance'/exp OR ‘multidrug resistan*’:ti,ab,kw OR ‘MDR resistan*’:ti,ab,kw OR ‘multi-drug resistan*’:ti,ab,kw OR ‘multiple drug resistan*’:ti,ab,kw OR 'beta lactam'/exp OR 'beta lactam*':ti,ab,kw OR ‘betalactam*’:ti,ab,kw OR ‘b-lactam*’:ti,ab,kw OR ‘blactam*’:ti,ab,kw OR 'beta lactamase'/exp OR 'extended spectrum beta lactamase'/exp OR ‘ESBL’:ti,ab,kw OR 'beta-lactam resistance'/exp OR 'penicillin resistance'/exp OR ‘penicillin*’:ti,ab,kw OR ‘PEN resistan*’:ti,ab,kw OR 'ampicillin'/exp OR 'ampicillin*':ti,ab,kw OR 'ampicillin resistance'/exp OR ‘Amp resistan*’:ti,ab,kw OR 'amoxicillin'/exp OR 'amoxicillin*':ti,ab,kw OR 'trimethoprim'/exp OR 'trimethoprim':ti,ab,kw OR 'trimethoprim resistance'/exp OR ‘TMP-resistan*’:ti,ab,kw OR 'sulfamethoxazole'/exp OR 'sulfamethoxazol*':ti,ab,kw OR 'cotrimoxazole'/exp OR 'cotrimoxazol*':ti,ab,kw OR ‘co trimoxazol*’:ti,ab,kw OR ’trimethoprim sulfamethoxazol*’:ti,ab,kw OR ‘sulfamethoxazole-trimethoprim’:ti,ab,kw OR ‘sulfamethoxazol-trimethoprim’:ti,ab,kw OR ‘sulfamethoxazole and trimethoprim’:ti,ab,kw OR ‘sulfamethoxazole plus trimethoprim’:ti,ab,kw OR ‘trimethoprim plus sulfamethoxazole’:ti,ab,kw OR ‘trimethoprim sulfamethoxazole combination’:ti,ab,kw OR ‘trimethoprim-sulfamethoxazole combination’:ti,ab,kw OR ‘trimethoprimsulfamethoxazole’:ti,ab,kw OR ‘trimetoprim-sulfa’:ti,ab,kw OR ‘trimetoprimsulfamethoxazole’:ti,ab,kw OR 'chloramphenicol'/exp OR 'chloramphenicol':ti,ab,kw OR 'chloramphenicol resistance'/exp OR ‘CA resistan*’:ti,ab,kw OR 'cephalosporin'/exp OR 'cephalosporin*':ti,ab,kw OR ‘cefalosporin*’:ti,ab,kw OR 'cephalosporin resistance'/exp OR 'ceftriaxone'/exp OR 'ceftriaxon*':ti,ab,kw OR 'cefotaxime'/exp OR 'cefotaxim*':ti,ab,kw OR 'cefotaxime resistance'/exp OR ‘CTX resistan*’:ti,ab,kw OR 'ceftazidime'/exp OR 'ceftazidim*':ti,ab,kw OR 'ceftazidime resistance'/exp OR ‘CAZ resistan*’:ti,ab,kw OR 'cefixime'/exp OR 'cefixim*':ti,ab,kw OR 'ciprofloxacin'/exp OR 'ciprofloxacin*':ti,ab,kw OR 'ciprofloxacin resistance'/exp OR ‘CIP resistan*’:ti,ab,kw OR ‘DCS’:ti,ab,kw OR 'ofloxacin'/exp OR 'ofloxacin':ti,ab,kw OR 'nalidixic acid'/exp OR 'nalidixic acid’:ti,ab,kw OR 'pefloxacin'/exp OR 'pefloxacin':ti,ab,kw OR 'gatifloxacin'/exp OR 'gatifloxacin':ti,ab,kw OR ‘fluoroquinolon*’:ti,ab,kw OR 'fluoroquinolone resistance'/exp OR ‘fluorinated quinolone resistan*’:ti,ab,kw OR ‘fluoro-quinolone resistan*’:ti,ab,kw OR ‘fluoroquinolones resistan*’:ti,ab,kw OR 'azithromycin'/exp OR 'azithromycin*':ti,ab,kw OR ‘macrolide'/exp OR ‘macrolide*':ti,ab,kw OR 'macrolide resistance'/exp OR 'tigecycline'/exp OR 'tigecycline’:ti,ab,kw OR 'temocillin'/exp OR 'temocillin*':ti,ab,kw OR ‘meropenem'/exp OR ‘meropenem':ti,ab,kw OR 'carbapenem'/exp OR 'carbapenem*':ti,ab,kw OR ‘carbapenem resistance'/exp OR 'colistin'/exp OR 'colistin':ti,ab,kw OR 'aztreonam'/exp OR 'aztreonam':ti,ab,kw OR 'sulfonamide'/exp OR 'sulfonamide*':ti,ab,kw OR 'sulphonamide*':ti,ab,kw OR ‘Perfloxacin*’:ti,ab,kw OR 'levofloxacin'/exp OR 'levofloxacin*':ti,ab,kw

**AND**

**CONCEPT: Non-typhoidal *Salmonella***

'Salmonella enterica serovar Enteritidis'/exp OR 'Salmonella enterica serovar Enteritidis':ti,ab,kw OR 'Salmonella Enteritidis':ti,ab,kw OR 'Salmonella enterica serovar Typhimurium'/exp OR 'Salmonella enterica serovar Typhimurium':ti,ab,kw OR ‘Salmonella typhimurium’:ti,ab,kw OR ‘nontyphoidal salmonella’:ti,ab,kw OR ‘non typhoidal salmonella’:ti,ab,kw OR ‘non typhi salmonella’:ti,ab,kw OR ‘non typhoidal’:ti,ab,kw OR ‘nontyphoidal’:ti,ab,kw OR ‘non typhi’:ti,ab,kw OR ‘salmonella infection*’:ti,ab,kw OR 'salmonellosis'/exp OR ‘salmonellosis’:ti,ab,kw OR ‘salmonelloses’:ti,ab,kw OR 'Salmonella'/exp OR 'Salmonella’:ti,ab,kw

**AND**

**CONCEPT: Invasive infection**

'bacteremia'/exp OR ‘bacteremia’:ti,ab,kw OR ‘bacteraemia’:ti,ab,kw OR ‘bacteriemia’:ti,ab,kw OR 'sepsis'/exp OR ‘sepsis’:ti,ab,kw OR ‘sepsis syndrome’:ti,ab,kw OR ‘septic disease’:ti,ab,kw OR 'septicemia'/exp OR ‘septicemia*’:ti,ab,kw OR ‘septicaemia*’:ti,ab,kw OR ‘invasive’:ti,ab,kw OR ‘extraintestinal-infection*’:ti,ab,kw OR ‘extra intestinal infection*’:ti,ab,kw OR ‘septi*’:ti,ab,kw OR ‘blood*’:ti,ab,kw OR ‘bloodstream*’:ti,ab,kw OR ‘bloodculture’:ti,ab,kw OR ‘blood-culture’:ti,ab,kw OR ‘hemoculture’:ti,ab,kw

**AND**

**CONCEPT: sub-Saharan Africa**

'Africa south of the Sahara'/exp OR ‘Sub-Saharan Africa’:ti,ab,kw OR ‘Subsaharan Africa’:ti,ab,kw OR ‘Sub Saharan Africa’:ti,ab,kw OR 'Central Africa'/exp OR 'Central Africa':ti,ab,kw OR 'Cameroon'/exp OR 'Cameroon':ti,ab,kw OR 'Central African Republic'/exp OR 'Central African Republic':ti,ab,kw OR 'Chad'/exp OR 'Chad':ti,ab,kw OR 'Congo'/exp OR 'Congo':ti,ab,kw OR 'Democratic Republic Congo'/exp OR 'Democratic Republic Congo':ti,ab,kw OR 'Equatorial Guinea'/exp OR 'Equatorial Guinea':ti,ab,kw OR 'Gabon'/exp OR 'Gabon':ti,ab,kw OR 'Sao Tome and Principe'/exp OR 'Sao Tome and Principe':ti,ab,kw OR ‘Eastern Africa’:ti,ab,kw OR ‘East Africa’:ti,ab,kw OR 'Burundi'/exp OR 'Burundi':ti,ab,kw OR 'Djibouti'/exp OR 'Djibouti':ti,ab,kw OR 'Eritrea'/exp OR 'Eritrea':ti,ab,kw OR 'Ethiopia'/exp OR 'Ethiopia':ti,ab,kw OR 'Kenya'/exp OR 'Kenya':ti,ab,kw OR 'Rwanda'/exp OR 'Rwanda':ti,ab,kw OR 'Somalia'/exp OR 'Somalia':ti,ab,kw OR 'South Sudan'/exp OR 'South Sudan':ti,ab,kw OR 'Sudan'/exp OR 'Sudan':ti,ab,kw OR 'Tanzania'/exp OR 'Tanzania':ti,ab,kw OR 'Uganda'/exp OR 'Uganda':ti,ab,kw OR ‘Southern Africa’:ti,ab,kw OR ‘South Africa’:ti,ab,kw OR 'Angola'/exp OR 'Angola':ti,ab,kw OR 'Botswana'/exp OR 'Botswana':ti,ab,kw OR 'Lesotho'/exp OR 'Lesotho':ti,ab,kw OR 'Malawi'/exp OR 'Malawi':ti,ab,kw OR 'Mozambique'/exp OR 'Mozambique':ti,ab,kw OR 'Namibia'/exp OR 'Namibia':ti,ab,kw OR 'South Africa'/exp OR 'South Africa':ti,ab,kw OR 'Swaziland'/exp OR 'Swaziland':ti,ab,kw OR 'Zambia'/exp OR 'Zambia':ti,ab,kw OR 'Zimbabwe'/exp OR 'Zimbabwe':ti,ab,kw OR ‘Western Africa’:ti,ab,kw OR ‘West Africa’:ti,ab,kw OR 'Benin'/exp OR 'Benin':ti,ab,kw OR 'Burkina Faso'/exp OR 'Burkina Faso':ti,ab,kw OR 'Cape Verde'/exp OR 'Cape Verde':ti,ab,kw OR 'Cote d`Ivoire'/exp OR 'Cote d`Ivoire':ti,ab,kw OR 'Gambia'/exp OR 'Gambia':ti,ab,kw OR 'Ghana'/exp OR 'Ghana':ti,ab,kw OR 'Guinea'/exp OR 'Guinea':ti,ab,kw OR 'Guinea-Bissau'/exp OR 'Guinea-Bissau':ti,ab,kw OR 'Liberia'/exp OR 'Liberia':ti,ab,kw OR 'Mali'/exp OR 'Mali':ti,ab,kw OR 'Mauritania'/exp OR 'Mauritania':ti,ab,kw OR 'Niger'/exp OR 'Niger':ti,ab,kw OR 'Nigeria'/exp OR 'Nigeria':ti,ab,kw OR 'Senegal'/exp OR 'Senegal':ti,ab,kw OR 'Sierra Leone'/exp OR 'Sierra Leone':ti,ab,kw OR 'Togo'/exp OR 'Togo':ti,ab,kw OR 'Comoros'/exp OR 'Comoros':ti,ab,kw OR 'Mayotte'/exp OR 'Mayotte':ti,ab,kw OR 'Madagascar'/exp OR 'Madagascar':ti,ab,kw OR 'Sahel'/exp OR 'Sahel':ti,ab,kw

### Ovid Embase - Research question 2: Antimicrobial therapy for invasive NTS infections

**CONCEPT: Antimicrobial treatment**

'therapy'/de OR 'drug therapy'/exp OR 'drug therapy':ti,ab,kw OR 'drug therapies':ti,ab,kw OR ‘drug treatment*’:ti,ab,kw OR ‘antibiotic therapy':ti,ab,kw OR ‘antibiotic therapies':ti,ab,kw OR ‘antibiotic treatment*’:ti,ab,kw OR ‘antibiotic management’:ti,ab,kw OR ‘antibacterial therapy’:ti,ab,kw OR ‘antibacterial therapies’:ti,ab,kw OR ‘anti-bacterial therapy’:ti,ab,kw OR ‘anti-bacterial therapies’:ti,ab,kw OR ‘antibacterial treatment*’:ti,ab,kw OR ‘anti-bacterial treatment*’:ti,ab,kw OR 'antiinfective agent'/exp OR 'antiinfective agent*':ti,ab,kw OR ‘anti-infective agent*’:ti,ab,kw OR ‘anti-bacterial agent*’:ti,ab,kw OR ‘antibacterial agent*’:ti,ab,kw OR ‘antibacterial drug*’:ti,ab,kw OR ‘antimicrobial agent*’:ti,ab,kw OR ‘antimicrobial compound*’:ti,ab,kw OR ‘antimicrobial drug*’:ti,ab,kw OR ‘antiseptic agent*’:ti,ab,kw OR 'therapy':ti,ab,kw OR 'therapies':ti,ab,kw OR ‘treatment*’:ti,ab,kw OR ‘management*’:ti,ab,kw OR ‘antibiotic*’:ti,ab,kw OR ‘antimicrobial’:ti,ab,kw

**AND**

**CONCEPT: Invasive infection**

'bacteremia'/exp OR ‘bacteremia’:ti,ab,kw OR ‘bacteraemia’:ti,ab,kw OR ‘bacteriemia’:ti,ab,kw OR 'sepsis'/exp OR ‘sepsis’:ti,ab,kw OR ‘sepsis syndrome’:ti,ab,kw OR ‘septic disease’:ti,ab,kw OR 'septicemia'/exp OR ‘septicemia*’:ti,ab,kw OR ‘septicaemia*’:ti,ab,kw OR ‘invasive’:ti,ab,kw OR ‘extraintestinal-infection*’:ti,ab,kw OR ‘extra intestinal infection*’:ti,ab,kw OR ‘septi*’:ti,ab,kw OR ‘blood*’:ti,ab,kw OR ‘bloodstream*’:ti,ab,kw OR ‘bloodculture’:ti,ab,kw OR ‘blood-culture’:ti,ab,kw OR ‘hemoculture’:ti,ab,kw

**AND**

**CONCEPT: Non-typhoidal *Salmonella***

'Salmonella enterica serovar Enteritidis'/exp OR 'Salmonella enterica serovar Enteritidis':ti,ab,kw OR 'Salmonella Enteritidis':ti,ab,kw OR 'Salmonella enterica serovar Typhimurium'/exp OR 'Salmonella enterica serovar Typhimurium':ti,ab,kw OR ‘Salmonella typhimurium’:ti,ab,kw OR ‘nontyphoidal salmonella’:ti,ab,kw OR ‘non typhoidal salmonella’:ti,ab,kw OR ‘non typhi salmonella’:ti,ab,kw OR ‘non typhoidal’:ti,ab,kw OR ‘nontyphoidal’:ti,ab,kw OR ‘non typhi’:ti,ab,kw OR ‘salmonella infection*’:ti,ab,kw OR 'salmonellosis'/exp OR ‘salmonellosis’:ti,ab,kw OR ‘salmonelloses’:ti,ab,kw OR 'Salmonella'/exp OR 'Salmonella’:ti,ab,kw

## Table S2. In- and exclusion criteria for both research questions.

| **Research questions** | **Population** | **Intervention** | **Comparison** | **Outcome** | **Study design** |
| --- | --- | --- | --- | --- | --- |
| 1. **AMR in invasive NTS infection in SSA** | NTS from normally sterile body sites from patients living in / travelling from SSA | Not applicable | Not applicable | Phenotypic antimicrobial susceptibility: see Additional File 2 for a list of which antimicrobials | - Interventional studies - Observational studies - Case series/ reports |
|  | - (Pooled with) intestinal isolates - (Pooled with) *Salmonella* (Para)Typhi - <10 NTS per country - Reported collection of NTS already fully described in another included study | Not applicable | Not applicable | - Susceptibility not reported per antibiotic agent - Only genotypic antibiotic susceptibility results | - Reviews - Experimental studies |
| 1. **Antimicrobial therapy for invasive NTS infections** | Patients with invasive NTS infection | Antimicrobial therapy regimen: antibiotic agent, route of administration, dose and duration | No or other antimicrobial therapy regimens: antibiotic agent, administration route, dose and duration | - Antimicrobial treatment efficacy: case fatality, fever clearance time, microbiological clearance, recurrence, fecal carriage and adverse effects - Recommended antimicrobial treatment regimens | - Guidelines - Opinion papers - Narrative/scoping/systematic   reviews and meta-analyses - Interventional studies - Observational studies |
|  | Patients with enteric fever or intestinal NTS infection |  |  | - Outcome not reported per therapeutic regimen - Outcome for <10 patients per therapeutic regimen | - Case series with outcome for <10 patients per therapeutic regimen & case reports, except if extensive literature review - Experimental studies - In vitro studies |

Legend: Inclusion criteria are presented in green, exclusion criteria are presented in red. Abbreviations: AMR : antimicrobial resistance ; NTS : non-typhoidal Salmonella ; SSA: sub-Saharan Africa

## List S2. Published articles from which no full text was found

### Research question 1: Antimicrobial resistance in invasive NTS infections in Sub-Saharan Africa

1. **Antibiotic sensitivity profiles of salmonella organisms isolated from presumptive typhoid patients in Zaria, northern Nigeria.
   Authors:** YKE Ibrahim; TA Adedare; JO Ehinmidu; 
   Afr J Med Med Sci, 34 (2), 109-14 Jun 2005
2. **Predominant bacterial agents of childhood septicaemia in Jos.
   Authors:** IA Angyo; ES Opkeh; SO Opajobi; 
   Niger J Med, 10 (2), 75-7 Apr-Jun 2001
3. **Septicemies a Salmonella au Centre Hospitalier National Yalgado Ouedraogo (CHN-YO) de Ouagadougou; Burkina Faso 
   Authors:** Sangare; L Sanou; I Ouedraogo-Traore; R Soudre; RB Samb;

### Research question 2: Antimicrobial therapy for invasive NTS infections

1. **Duration of antimicrobial therapy for non-typhoid Salmonella bacteremia in healthy children
   Authors:** MH Yen; YC Huang; CH Chiu; TY Lin;
   J Microbiol Immunol Infect, 35 (2), 94-8 Jun 2002
2. **Salmonella-a new threat to neonates.
   Authors:** SA Begum; AB Lutfor; AH Mollah; MK Hasan; S Ahmed ; M Akhter; NM Salauddin; 
   Mymensingh Med J, 16 (2 Suppl), S15-18 Jul 2007

## Figure S1. Sensitivity analysis for multidrug resistance (MDR, panel A), third generation cephalosporins (C3G-resistance, panel B) & fluoroquinolone non-susceptibility (FQNS, panel C)


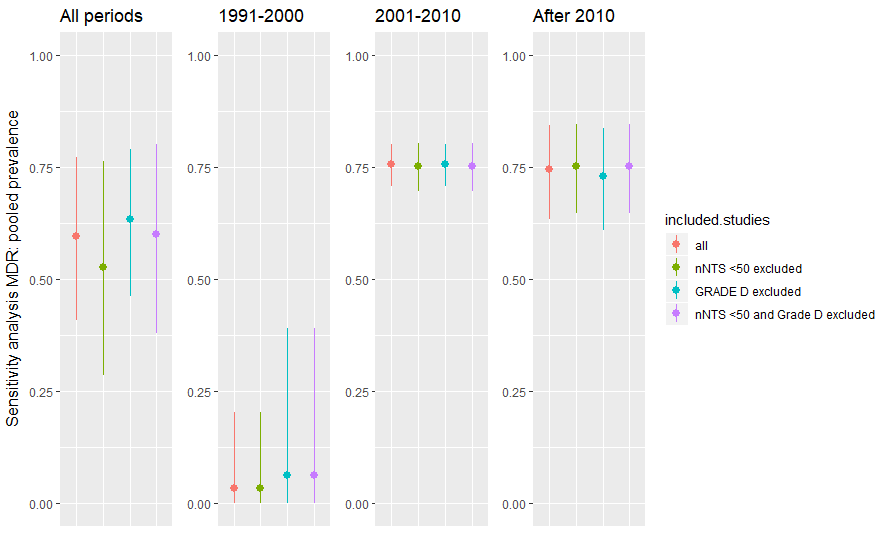


**A**


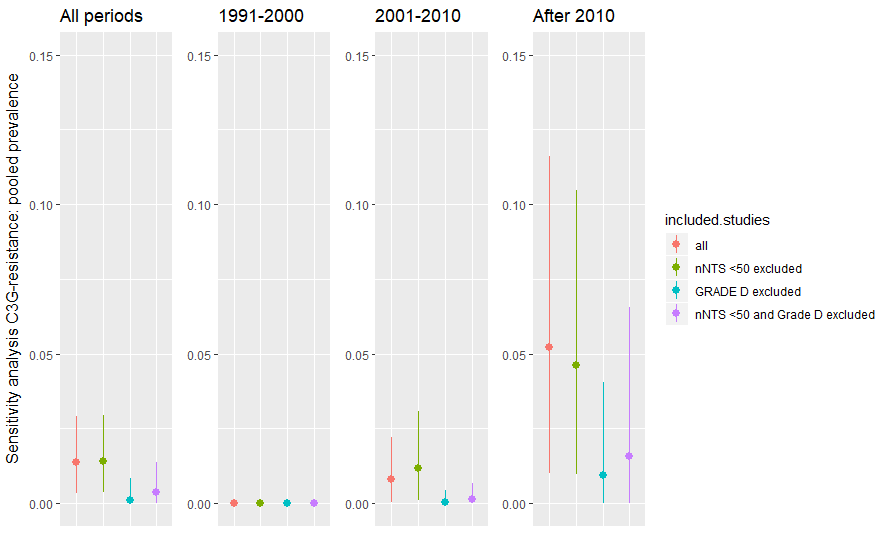


**B**


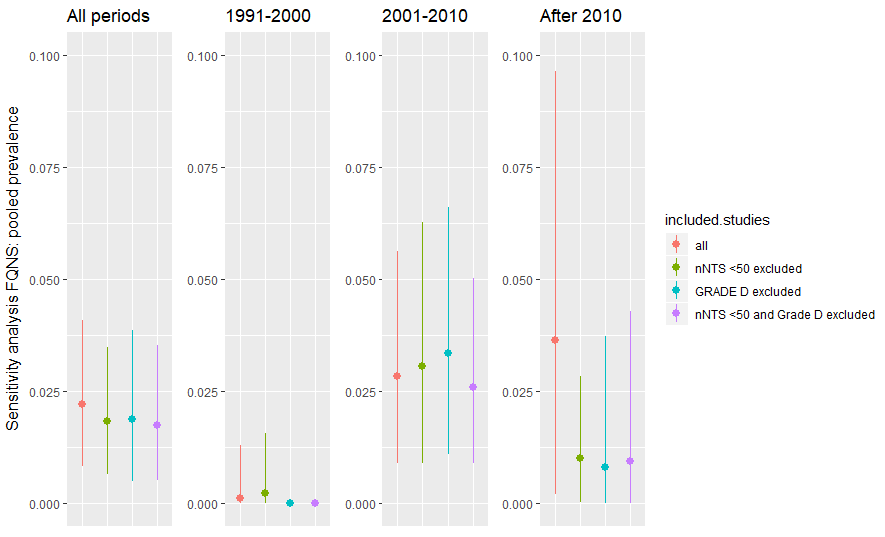


**C**

Legend: Graphic representation of the sensitivity of the pooled proportions to the inclusion of studies with a low quality grade or sample size; abbreviations: nNTS<50: studies in which the antibiotic susceptibility testing results were reported for fewer than 50 NTS isolates

## Figure S2. Meta-analysis and forest plots of multidrug resistance, third generation cephalosporin resistance and fluoroquinolone non-susceptibility according to NTS serotype

### Figure S2A. Meta-analysis according to NTS-serotype: Multidrug resistance
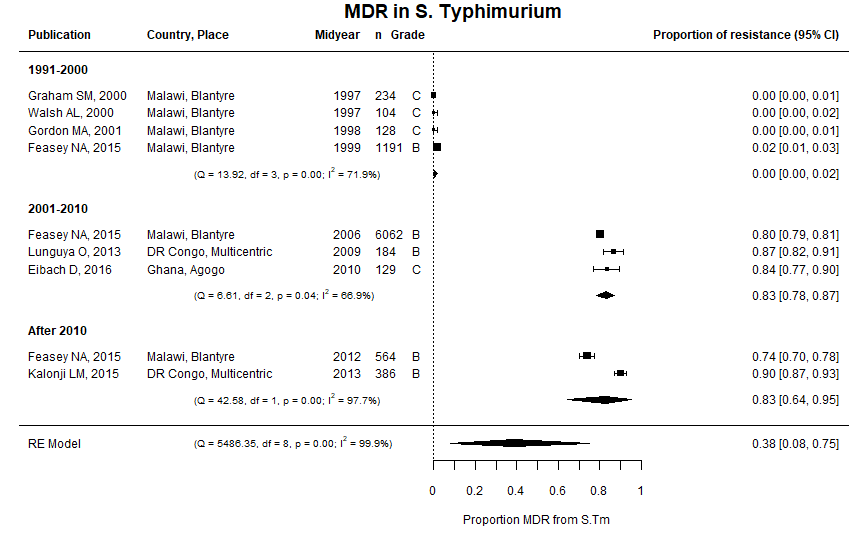


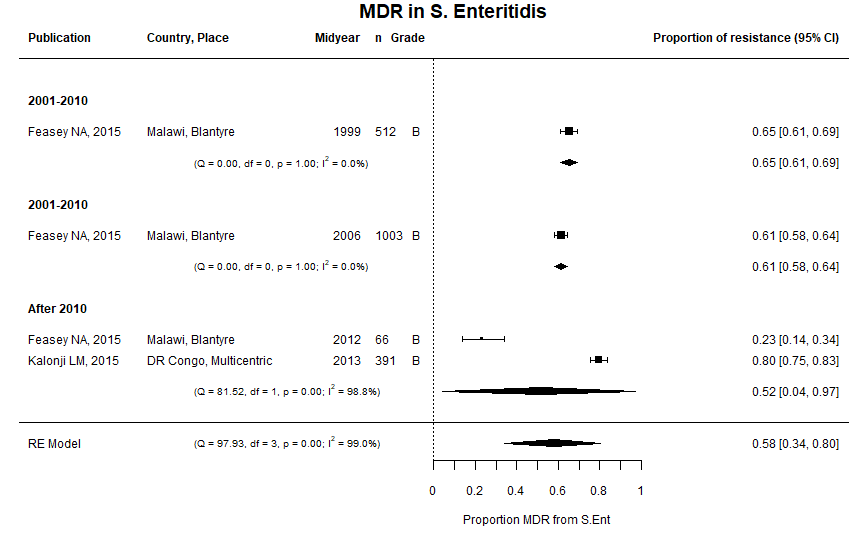


Legend: Each publication is identified by its first author and year of publication. Studies are ranked by the midyear of the study period during which the NTS were isolated. The grade represents the study quality and was assessed based on the MICRO checklist. [11] Abbreviations: MDR: multidrug resistance; S. Tm: *Salmonella* Typhimurium; S. Ent: *Salmonella* Enteritidis; RE model: Random effects model ; df: degrees of freedom ; 95% CI: 95% confidence inter

### Figure S2B. Meta-analysis according to NTS-serotype: third generation cephalosporin resistance


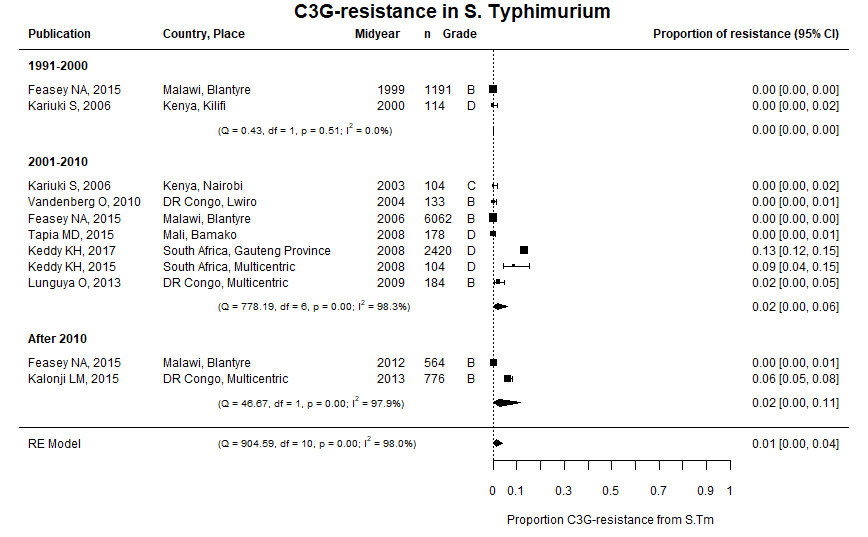

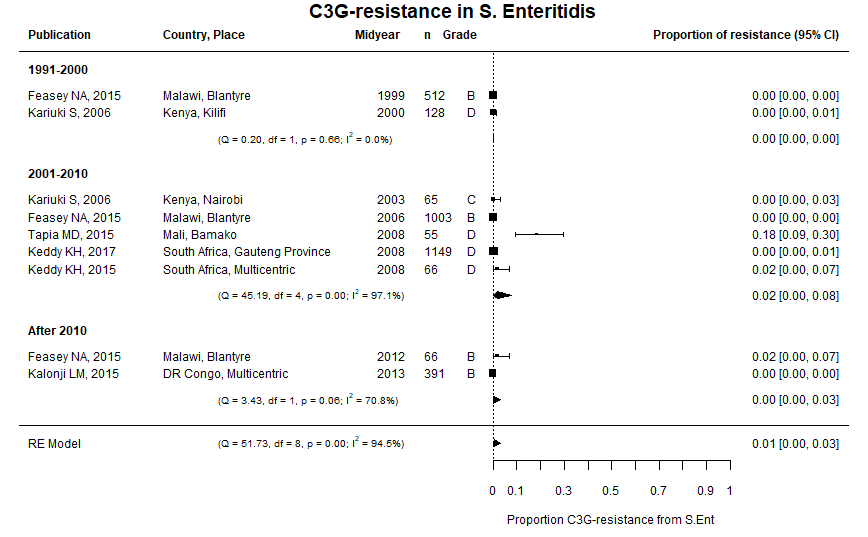
Legend: Each publication is identified by its first author and year of publication. Studies are ranked by the midyear of the study period during which the NTS were isolated. The grade represents the study quality and was assessed based on the MICRO checklist. [11] Abbreviations: C3G-resistance: third generation cephalosporin resistance; S. Tm: *Salmonella* Typhimurium; S. Ent: *Salmonella* Enteritidis; RE model: Random effects model ; df: degrees of freedom ; 95% CI: 95% confidence interval

### Figure S2C. Meta-analysis according to NTS-serotype: fluoroquinolone non-susceptibility


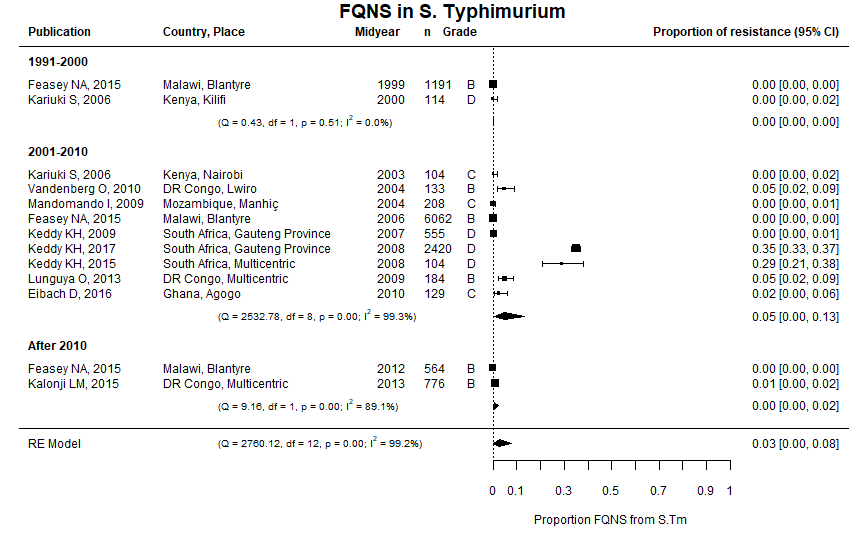


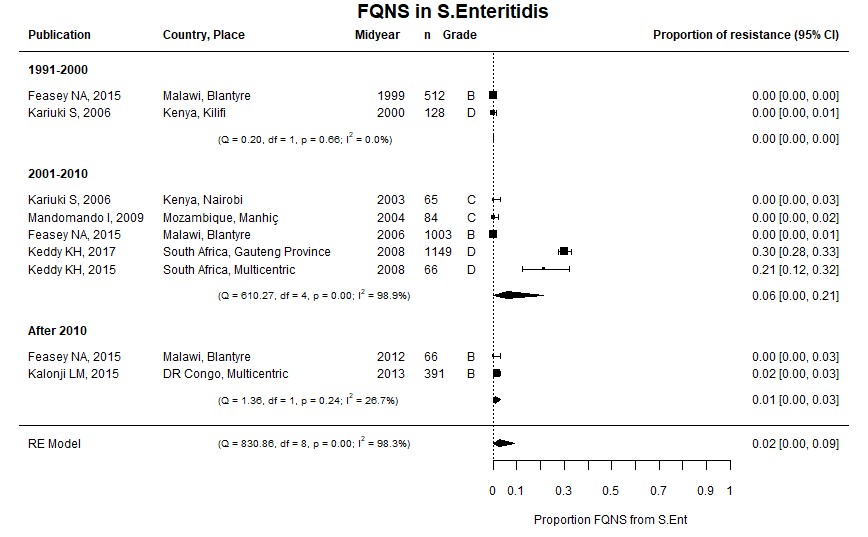


Legend: Each publication is identified by its first author and year of publication. Studies are ranked by the midyear of the study period during which the NTS were isolated. The grade represents the study quality and was assessed based on the MICRO checklist. [11] Abbreviations: FQNS: fluoroquinolone non-susceptibility; S. Tm: *Salmonella* Typhimurium; S. Ent: *Salmonella* Enteritidis; RE model: Random effects model ; df: degrees of freedom; 95% CI: 95% confidence interval

## Table S3. Meta-regression to identify moderators of the pooled proportions of multidrug resistance (MDR), third generation cephalosporin resistance (C3G-resistance) and fluoroquinolone non-susceptibility (FQNS)

| **MDR** | **N studies** | **Tau2** | **I2** | **Q** | **df** | **p-val Q** | **p-val moderator** |
| --- | --- | --- | --- | --- | --- | --- | --- |
| No moderators | 11 | 0.1729 | 99.8% | 2916 | 10 | <0.001 | NA |
| Midyear (continuous) | 11 | 0.0359 | 98.9% | 1061 | 9 | <0.001 | <0.001 |
| Period | 11 | 0.0204 | 96.2% | 222 | 8 | <0.001 | <0.001 |
| Period 2 |  |  |  |  |  |  | 0.02 |
| Period 3 |  |  |  |  |  |  | <0.001 |
| Period 4 |  |  |  |  |  |  | <0.001 |
| Study duration | 11 | 0.2075 | 99.7% | 1771 | 7 | <0.001 | 0.72 |
| Study design | 11 | 0.1924 | 99.8% | 2905 | 9 | <0.001 | 0.95 |
| Level of study setting | 8 | 0.0469 | 99.4% | 2015 | 6 | <0.001 | 0.24 |
| Age category study population | 11 | 0.1382 | 99.7% | 2635 | 9 | <0.001 | 0.06 |
| HIV prevalence study population | 3 | 0.5710 | 99.8% | 425 | 1 | <0.001 | 0.58 |
| Source (only blood or not) | 11 | 0.1715 | 99.8% | 2880 | 9 | <0.001 | 0.29 |
| Grade | 11 | 0.1366 | 99.8% | 2675 | 8 | <0.001 | 0.10 |

| **C3G - resistance** | **N studies** | **Tau2** | **I2** | **Q** | **df** | **p-val Q** | **p-val moderator** |
| --- | --- | --- | --- | --- | --- | --- | --- |
| No moderators | 27 | 0.0156 | 97.5% | 1383 | 26 | <0.001 | NA |
| Midyear (continuous) | 27 | 0.0120 | 96.7% | 1075 | 25 | <0.001 | 0.005* |
| Period | 27 | 0.0126 | 96.5% | 1216 | 24 | <0.001 | 0.02* |
| Period 2 |  |  |  |  |  |  | 0.55 |
| Period 3 |  |  |  |  |  |  | 0.13 |
| Period 4 |  |  |  |  |  |  | 0.006* |
| Study duration | 27 | 0.0167 | 97.4% | 1333 | 24 | <0.001 | 0.84 |
| Study design | 27 | 0.0152 | 97.4% | 1346 | 25 | <0.001 | 0.19 |
| Level of study setting | 23 | 0.0157 | 97.8% | 1268 | 20 | <0.001 | 0.17 |
| Age category study population | 25 | 0.0176 | 97.8% | 1340 | 22 | <0.001 | 0.91 |
| HIV prevalence study population | 5 | 0.0284 | 96.4% | 40 | 3 | <0.001 | 0.30 |
| Source (only blood or not) | 27 | 0.0162 | 97.4% | 1376 | 25 | <0.001 | 0.62 |
| Grade | 27 | 0.0146 | 96.7% | 795 | 24 | <0.001 | 0.17 |

| **FQNS** | **N studies** | **Tau2** | **I2** | **Q** | **df** | **p-val Q** | **p-val moderator** |
| --- | --- | --- | --- | --- | --- | --- | --- |
| No moderators | 30 | 0.0175 | 97.8% | 3703 | 29 | <0.001 | NA |
| Midyear (continuous) | 30 | 0.0171 | 97.7% | 3348 | 28 | <0.001 | 0.19 |
| Period | 30 | 0.0162 | 97.4% | 3288 | 27 | <0.001 | 0.14 |
| Study duration | 30 | 0.0165 | 97.4% | 3314 | 27 | <0.001 | 0.20 |
| Study design | 30 | 0.0176 | 97.8% | 3703 | 28 | <0.001 | 0.35 |
| Level of study setting | 26 | 0.0176 | 98.1% | 3658 | 23 | <0.001 | 0.11 |
| Age category study population | 28 | 0.0175 | 97.8% | 3465 | 25 | <0.001 | 0.18 |
| HIV prevalence study population | 6 | 0.0629 | 99.0% | 547 | 4 | <0.001 | 0.62 |
| Source (only blood or not) | 30 | 0.0197 | 97.7% | 3550 | 28 | <0.001 | 0.48 |
| Grade | 30 | 0.0183 | 97.5% | 2522 | 27 | <0.001 | 0.68 |
| DCS defined | 30 | 0.0178 | 97.7% | 3536 | 28 | <0.001 | 0.45 |

Legend : DCS : decreased ciprofloxacin susceptibility
